# Linking Creatinine‐to‐Body Weight Ratio With Diabetes Incidence: A Multiethnic Malaysian Cohort Study

**DOI:** 10.1111/1753-0407.70039

**Published:** 2025-01-22

**Authors:** Noraidatulakma Abdullah, Ying‐Xian Goh, Aisyatul Najihah Khuzaimi, Azwa Shawani Kamalul Arifin, Nurul Ain Mhd Yusuf, Nazihah Abd Jalal, Norliza Ismail, Nurul Faeizah Husin, Mohd Arman Kamaruddin, Rahman Jamal

**Affiliations:** ^1^ UKM Medical Molecular Biology Institute (UMBI) Universiti Kebangsaan Malaysia Kuala Lumpur Malaysia

**Keywords:** body fat mass, body weight, creatinine, diabetes, multiethnic population

## Abstract

**Background:**

Emerging evidence suggests that the creatinine‐to‐body weight (Cre/BW) ratio is a predictor for incident diabetes in the Asian population. This study examined the association between Cre/BW ratio and incident diabetes, as well as the relationship between Cre/BW ratio and skeletal muscle and body fat mass in a multiethnic Malaysian cohort.

**Methods:**

A total of 13 047 eligible participants were selected from 119 560 The Malaysian Cohort participants. Of these, 750 who developed diabetes were selected as cases, while 3750 controls were chosen randomly from healthy participants. This nested case–control study included 4500 eligible participants from The Malaysian Cohort, with a 1:5 case‐to‐control ratio. Participants were stratified into four groups based on Cre/BW ratio quartiles. The Cox proportional hazards model evaluated the effect of Cre/BW ratio on developing incident diabetes. The association between Cre/BW ratio and body composition was assessed using the Pearson correlation coefficient.

**Results:**

Of the 13 047 eligible participants followed up over 5.3 years, 5.75% (*n* = 750) developed diabetes. Diabetes incidence decreased with increasing Cre/BW ratios. The Cre/BW ratio was inversely correlated with diabetes risk (HR: 0.403, 95% CI: 0.315–0.515, *p* < 0.001). Additionally, males and Indians had a higher risk of developing incident diabetes. A significant correlation was observed between Cre/BW ratio and body fat mass (*p* < 0.001).

**Conclusions:**

This study reveals an inverse association between the Cre/BW ratio and incident diabetes. It also found a significant moderate correlation between the Cre/BW ratio and body fat mass.

## Graphical abstract

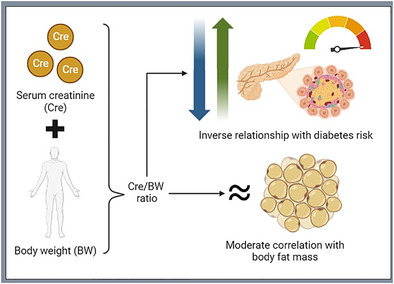

**Summary**

- Creatinine‐to‐body weight (Cre/BW) ratio is negatively associated with diabetes risk, regardless of gender and ethnicity.
- From the Cre/BW ratio's perspective, males and Indians have a higher diabetes risk than their counterparts.
- The Cre/BW ratio shows a moderate but significant correlation with body fat mass.

## 1 Introduction

Diabetes is a complex disease attributed to the interplay of genetic, environmental, and physiological risk factors [1]. According to the World Health Organization (WHO), the Western Pacific and Southeast Asia (SEA) regions have the highest prevalence of diabetes worldwide, with 206 million and 90 million diabetes cases, respectively [2]. The Western Pacific and SEA regions are home to members of the Association of Southeast Asian Nations (ASEAN), many of which are low‐ and middle‐income countries (LMICs), such as Indonesia, Lao People's Democratic Republic (PDR), Myanmar, Philippines, Thailand, and Malaysia. In the LMICs, diabetes prevalence has been rising faster than in high‐income countries [3], where over three in four adults in LMICs are living with diabetes [2]. Malaysia, as a middle‐income country, has the topmost diabetes prevalence among ASEAN member states [2], with an estimated 7 million Malaysian adults aged 18 and older expected to be affected by 2025 [4]. Also, the prevalence of diabetes is ever‐increasing in Malaysia, with 13.4% in 2015 to 18.3% in 2019 [4].

Recent studies have shown that the serum creatinine‐to‐body weight (Cre/BW) ratio is highly related to the incidence of diabetes among East Asians, particularly in the Japanese [5] and Chinese populations [6, 7]. However, they did not correlate the Cre/BW ratio to a specific body composition component. Serum creatinine, a waste product of muscle, has been reported to be associated with total skeletal muscle mass (SMM) [8, 9]. Also, serum creatinine has been proven to be an inexpensive, readily accessible, and stable alternative indicator of muscle quality in individuals with normal renal functions [8]. Studies have shown that the muscle protein metabolism and skeletal muscle atrophy of diabetic patients are abnormal—deteriorating more rapidly than a non‐diabetic patient [10, 11]. The reduction in skeletal muscle eventually leads to reduced systemic glucose uptake [10], hence causing insulin resistance [12]. Taking these together, the Cre/BW ratio may serve as a valuable predictor of diabetes by reflecting lean body mass. While body mass index (BMI), waist circumference (WC), and waist‐to‐height ratio provide useful information on obesity and fat accumulation, they do not offer insights into muscle mass, which plays a crucial role in glucose metabolism [13]. BMI, for instance, is a general indicator of body weight relative to height but does not distinguish between muscle and fat, which can lead to misinterpretation, particularly in athletes with high muscle mass and high BMI but low diabetes risk, and in the elderly with age‐related sarcopenia. WC and waist‐to‐height ratio are useful for assessing abdominal obesity, a condition closely linked to metabolic syndrome and diabetes, but they still fail to account for muscle mass, which can act as a protective factor against diabetes.

In addition, although Cre/BW ratio is found to be highly related to the incidence of diabetes in the Japanese and Chinese populations, this relationship has yet to be elucidated in other Asian populations such as among the Malays and Indians. Thus, this study was aimed to address these gaps by assessing the association between Cre/BW ratio and diabetes, as well as Cre/BW ratio and body composition in the multiethnic Malaysian population.

## 2 Methods

### 2.1 Study Population and Design

This is a nested case–control study with a ratio of 1:5 in terms of cases to controls, that was derived from The Malaysian Cohort (TMC) study. TMC is the population‐based prospective cohort study in Malaysia that has cumulatively recruited 119 560 participants aged 35–70 years starting from 2007 [14]. TMC has successfully followed up 52.1% of its baseline participants using both telephone interviews and physical follow‐ups [15]. This study was approved by the institutional review and ethics board of the Universiti Kebangsaan Malaysia (Project Code: FF‐205‐2007).

**FIGURE 1:**
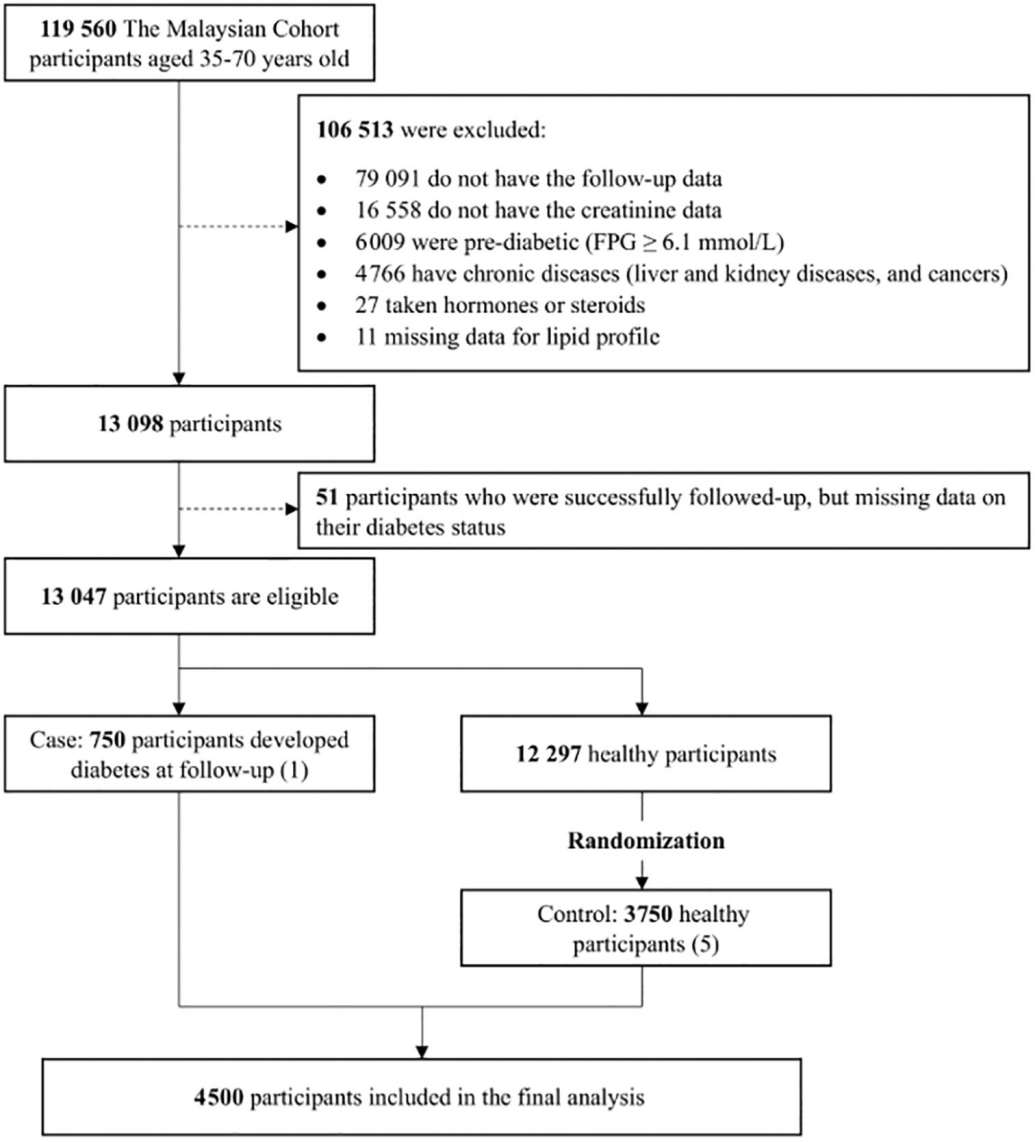
Flowchart of study participants and design.

In this current study, participants were chosen if they had completed the baseline and follow‐up assessment, which averaged a period of 4–6 years. Cases were those with no diabetes at baseline and later developed diabetes during follow‐up, while controls were those who did not have diabetes at follow‐up. Those who have no follow‐up data, missing covariate data, taking or having taken steroids and hormones, have pre‐diabetes and diabetes at baseline based on fasting plasma glucose (FPG) and self‐reported with medication, and those with cancers, chronic liver diseases, or kidney diseases at baseline were excluded from this study [5, 6, 7]. After exclusion, only 13 047 participants remained for further analysis (Figure 1).

Of 13 047 participants, only 750 participants fulfilled the requirement of cases, while 3750 controls were randomly selected from 12 297 healthy participants. The final sample size for this nested case–control study was 4500 participants, recruited between December 22, 2010 and December 31, 2020 (Figure 1).

### 2.2 Data Collection and Measurements

Data were collected via a questionnaire and various measurements [14]. The questionnaire consists of queries relating to demographics (age, gender, ethnicity), lifestyle (smoking, alcohol consumption), and medical history. The interviews at baseline and follow‐up were conducted by trained staff members. Smoking status was classified as a smoker and non‐smoker, while drinking status was classified as a drinker and non‐drinker.

Biochemical and biophysical measurements were taken on the day of the visit after overnight fasting. Biochemical parameters, such as FPG, total cholesterol (TC), high‐density lipoprotein cholesterol (HDL‐C), low‐density lipoprotein cholesterol (LDL‐C), triglyceride (TG), and serum creatinine (Cre) were measured by the Roche cobas 6000 analyzer, while hemoglobin A1c (HbA1c) was measured by the Bio‐Rad Variant II Turbo hemoglobin testing system [16]. All biochemical analyses were conducted at our ISO 15189:2012‐accredited laboratory. Prediabetes and incident diabetes were defined as FPG ≥ 6.1 mmol/L and FPG ≥ 7.0 mmol/L and/or self‐reported diabetes with medications during the follow‐up period, respectively.

As for biophysical measurements, weight and height were measured using the SECA weight scale (SECA, Germany) and the Harpenden stadiometer (Holtain Limited, UK), respectively, while the waist and hip circumference were measured using the SECA meter scale. Blood pressure (BP) parameters were assessed using the OMRON digital monitor model HEM‐907 (Omron, Japan). Body fat mass (BFM) and SMM were estimated using the InBody 720 bioelectric impendence analyzer (Biospace, South Korea). The BMI was calculated by dividing weight (kg) by the square of height (meters). The BMI (kg/m^2^) was later categorized into four groups according to the Asian cut‐off [17]. In addition, the waist‐to‐hip ratio (WHR) was calculated by dividing WC (cm) by hip circumference (cm). Abdominal obesity was defined as the WC > 102 cm (for men) and > 88 cm (for women). The waist‐to‐height ratio is calculated by dividing WC by height, while the Cre/BW ratio is determined by dividing the serum creatinine level (μmol/L) by body weight (kg). The Cre/BW ratio was subsequently stratified into four quartiles.

### 2.3 Statistical Analysis

Participants were stratified based on the quartiles of baseline Cre/BW ratios, where quartile 1 (Q1) is the lowest 25th percentile and Q4 is the highest 25th percentile of the Cre/BW ratio. Continuous and categorical data were presented as mean with standard deviation (SD) and frequency (percentage), respectively. A chi‐square test and a one‐way analysis of variance (ANOVA) test were performed subsequently. The risk of Cre/BW ratios to diabetes was estimated via hazard ratio (HR), and its 95% confidence interval (CI) using the Cox proportional hazards model. Model 1 is not adjusted, model 2 is adjusted for age, gender, and ethnicity, while model 3 is adjusted for age, gender, ethnicity, height, FPG, SBP, DBP, TC, HDL‐C, LDL‐C, TG, HbA1c, drinking status, and smoking status [5, 6, 7]. Subsequently, Kaplan–Meier analysis and log‐rank tests were performed based on gender and ethnicity to investigate the association between Cre/BW quartiles and incident diabetes. Bonferroni correction was used to counteract the multiple comparisons error in the log‐rank test. A Spearman correlation analysis was carried out to determine the correlation of the Cre/BW ratio to BFM and SMM. All tests were performed using the statistical package for the social sciences (SPSS) v22.0, and *p* < 0.05 on both sides was statistically significant.

## 3 Results

This study included a total of 4500 participants (750 cases and 3750 controls) after considering the exclusion criteria (Figure 1). Table 1 shows the baseline characteristics of the study participants, stratified based on the quartiles of Cre/BW ratio. The mean age of the study population is 48.1 (SD: 7.6) years and the age is significantly increased accordingly to Cre/BW ratio quartiles. We observed a significant downward trend for weight, BMI, and WC across the quartiles of the Cre/BW ratio (*p* < 0.001). In addition, the significant downward trend across quartile of Cre/BW ratio was observed even when the analyses were stratified by gender (Table S1) and ethnicity (Table S2) (*p* < 0.001).

**TABLE 1:** Baseline characteristics of participants according to the quartiles of Cre/BW ratios. The baseline characteristics that further stratified based on gender and ethnicities can be found in Tables S1 and S2.

Cre/BW	Overall	Q1 (*n* = 1121) (< 0.8642)	Q2 (*n* = 1087) (0.8642 to < 1.0267)	Q3 (*n* = 1194) (1.0267 to < 1.2332)	Q4 (*n* = 1098) (≥ 1.2332)	*p*
Age (years)	48.11 ± 7.59	46.44 ± 7.02	47.45 ± 7.42	48.3 ± 7.38	50.25 ± 8.02	< 0.001*
Gender						< 0.001*
Male	1850 (41.1)	179 (9.7)	327 (17.7)	540 (29.2)	804 (43.5)	
Female	2650 (58.9)	942 (35.5)	760 (28.7)	654 (24.7)	294 (11.1)	
Ethnicity						< 0.001*
Malay	1507 (33.5)	385 (25.5)	334 (22.2)	399 (26.5)	389 (25.8)	
Chinese	1743 (38.7)	413 (23.7)	461 (26.4)	493 (28.3)	376 (21.6)	
Indian	800 (17.8)	231 (28.9)	198 (24.8)	190 (23.8)	181 (22.6)	
Others	450 (10)	92 (20.4)	94 (20.9)	112 (24.9)	152 (33.8)	
Height (cm)	160.28 ± 8.45	158.84 ± 7.95	159.83 ± 8.47	160.44 ± 9.09	162.02 ± 7.89	< 0.001*
Weight (kg)	66.2 ± 13.62	74.03 ± 15.31	66.53 ± 12.37	63.28 ± 11.79	61.04 ± 10.97	< 0.001*
BMI (kg/m^2^)	25.68 ± 4.53	29.17 ± 5.02	25.94 ± 3.69	24.48 ± 3.49	23.18 ± 3.39	< 0.001*
BMI classification (kg/m^2^)						< 0.001*
Underweight (< 18.5)	136 (3)	3 (2.2)	10 (7.4)	32 (23.5)	91 (66.9)	
Normal (18.5–22.9)	1117 (24.8)	100 (9)	237 (21.2)	366 (32.8)	414 (37.1)	
Overweight (23.0–27.4)	1830 (40.7)	331 (18.1)	468 (25.6)	554 (30.3)	477 (26.1)	
Obese (> 27.5)	1417 (31.5)	687 (48.5)	372 (26.3)	242 (17.1)	116 (8.2)	
Waist‐to‐hip ratio	0.85 ± 0.08	0.85 ± 0.08	0.84 ± 0.08	0.85 ± 0.08	0.86 ± 0.08	0.001*
Waist circumference (cm)	83.92 ± 11.6	90.12 ± 12.39	84.07 ± 10.78	81.85 ± 10.43	79.68 ± 10.01	< 0.001*
Abdominal obesity						< 0.001*
Yes	820 (18.2)	525 (46.8)	186 (17.1)	88 (7.4)	21 (1.9)	
No	3680 (81.8)	596 (53.2)	901 (82.9)	1106 (92.6)	1077 (98.1)	
Systolic blood pressure (mmHg)	127.95 ± 18.61	128.12 ± 18.58	127.44 ± 18.05	127.61 ± 18.28	128.65 ± 19.5	0.413
Diastolic blood pressure (mmHg)	76.69 ± 11.05	77.5 ± 11.25	76.86 ± 11.07	76.02 ± 10.48	76.43 ± 11.36	0.010*
Fasting plasmid glucose (mmol/L)	5.27 ± 0.41	5.26 ± 0.41	5.26 ± 0.39	5.28 ± 0.42	5.3 ± 0.41	< 0.001*
HbA1c (%)	5.52 ± 0.11	5.53 ± 0.12	5.52 ± 0.12	5.52 ± 0.1	5.51 ± 0.1	0.049*
Total cholesterol (mmol/L)	5.59 ± 1.04	5.47 ± 1.05	5.59 ± 1.04	5.67 ± 1.01	5.63 ± 1.05	< 0.001*
HDL‐C (mmol/L)	1.44 ± 0.41	1.43 ± 0.38	1.47 ± 0.41	1.44 ± 0.42	1.4 ± 0.41	0.001*
LDL‐C (mmol/L)	3.54 ± 0.94	3.44 ± 0.94	3.51 ± 0.93	3.59 ± 0.91	3.6 ± 0.97	< 0.001*
Triglyceride (mmol/L)	1.44 ± 0.91	1.37 ± 0.88	1.41 ± 0.84	1.48 ± 0.97	1.48 ± 0.93	0.007*
Serum creatinine (μmol/L)	68.86 ± 18.15	54.28 ± 10.86	63.01 ± 11.86	70.81 ± 13.35	87.43 ± 17.22	< 0.001*
Smoking status						< 0.001*
Yes	982 (21.8)	104 (10.6)	186 (18.9)	284 (28.9)	408 (41.5)	
No	3518 (78.2)	1017 (28.9)	901 (25.6)	910 (25.9)	690 (19.6)	
Drinking status						< 0.001*
Yes	207 (4.6)	29 (14)	46 (22.2)	55 (26.6)	77 (37.2)	
No	3556 (79)	825 (23.2)	865 (24.3)	986 (27.7)	880 (24.7)	
Unknown	737 (16.4)	267 (36.2)	176 (23.9)	153 (20.8)	141 (19.1)	
Incident diabetes						< 0.001*
Yes	750 (16.7)	275 (36.7)	180 (24)	154 (20.5)	141 (18.8)	
No	3750 (83.3)	846 (22.6)	907 (24.2)	1040 (27.7)	957 (25.5)	

*Note:* Data are given as mean ± standard deviation (SD), or frequency (percentage), as appropriate.

Abbreviations: BMI, body mass index; Cre/BW, creatinine‐to‐body weight; HbA1c, hemoglobin A1c; HDL‐C, high‐density lipoprotein cholesterol; LDL‐C, low‐density lipoprotein cholesterol.

* *p* < 0.05.

**FIGURE 2:**
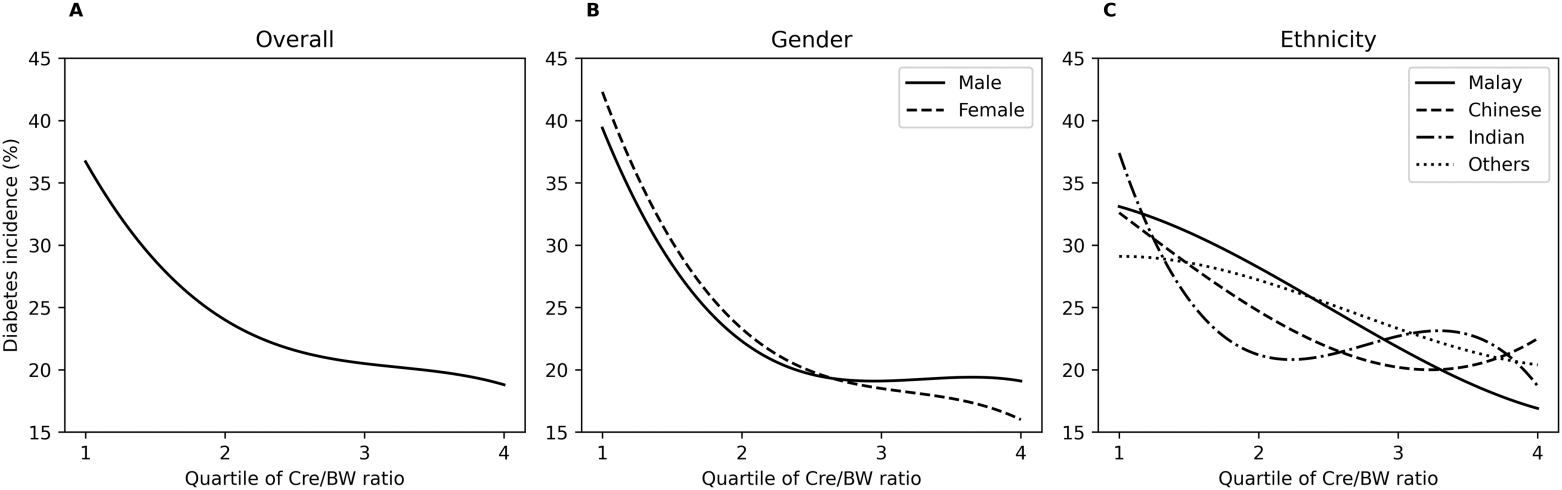
The (A) inverse relationship between Cre/BW quartiles and incident diabetes, further stratified by (B) gender and (C) ethnicity.

We observed an inverse relationship between diabetes incidence and the Cre/BW ratio, where the incidence of diabetes decreased with increasing Cre/BW ratio quartiles, regardless of gender and ethnicity (Figure 2). However, we did not find any significant difference in the risk of developing diabetes between males and females. In contrast, among ethnicities, Indians exhibited the highest risk of developing diabetes based on Cre/BW ratio quartiles.

**FIGURE 3:**
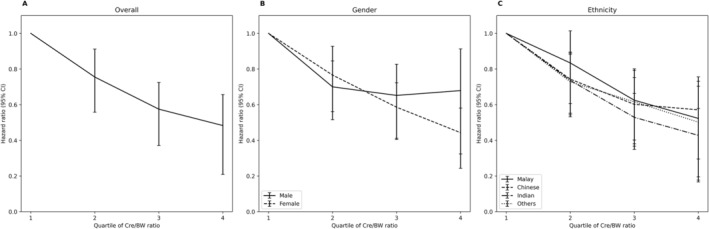
The relationship between Cre/BW ratio and incident diabetes in adjusted proportional hazards model 3, stratified based on gender and ethnicities. Additional information for both unadjusted and adjusted models that can be found in Tables [Link], [Link], [Link]; (A) The overall model is adjusted for age, gender, ethnicity, WHR, HDL‐C, TG, and SBP; (B) For males, the model is adjusted for age, ethnicity, WHR, TG, FPG, and BMI. For females, the model is adjusted for age, ethnicity, TG, and SBP; (C) Across different ethnicities, all models were adjusted for age, gender, HDL‐C, and DBP. Additionally, models for Malays and other ethnicities include an extra adjustment for WC.

A Cox proportional hazard model was used to assess the Cre/BW ratio's effect on the risk of incident diabetes. The effect sizes (HR and 95% CI) were presented in Figure 3 and Table S3. In general, we found a significant (*p* < 0.001) association between Cre/BW ratio and incident diabetes in both models 1 (crude) and 3 (adjusted for age, gender, ethnicity, WHR, HDL‐C, TG, and SBP). More details can be found in Table S3. Significant inversed associations between the Cre/BW ratio and the incidence of diabetes were also observed across models 1 to 3 (Figure 3 and Table S3), suggesting that a higher Cre/BW ratio indicated a lower incidence risk of diabetes (*p* < 0.05). In model 3, we found that the participants in Q4 had at least a 50% decreased risk in the odds of developing diabetes compared to those in Q1 (HR: 0.483, 95% CI: 0.309–0.757, *p* = 0.001). Similar downward trends with increasing Cre/BW ratio quartile were also observed across different models, stratified by gender (Table S4) and ethnicity (Table S5). Further analysis on interaction between ethnicity and Cre/BW ratio indicated significant inversed relationship (Table S7). To compare different indices in predicting diabetes incidence, we also assessed the relationship between two commonly used indices—BMI and waist‐to‐height ratio—and diabetes incidence. A positive association was observed between both BMI and diabetes incidence, as well as waist‐to‐height ratio and diabetes (Table S6). However, the crude and adjusted HRs for BMI were close to one, indicating that BMI is a weaker predictor of diabetes incidence compared to the Cre/BW ratio (Table S6). As for the waist‐to‐height ratio, the unusually high HR suggests considerable uncertainty in the precise estimate of the effect size, which should be interpreted with caution (Table S6).

**FIGURE 4:**
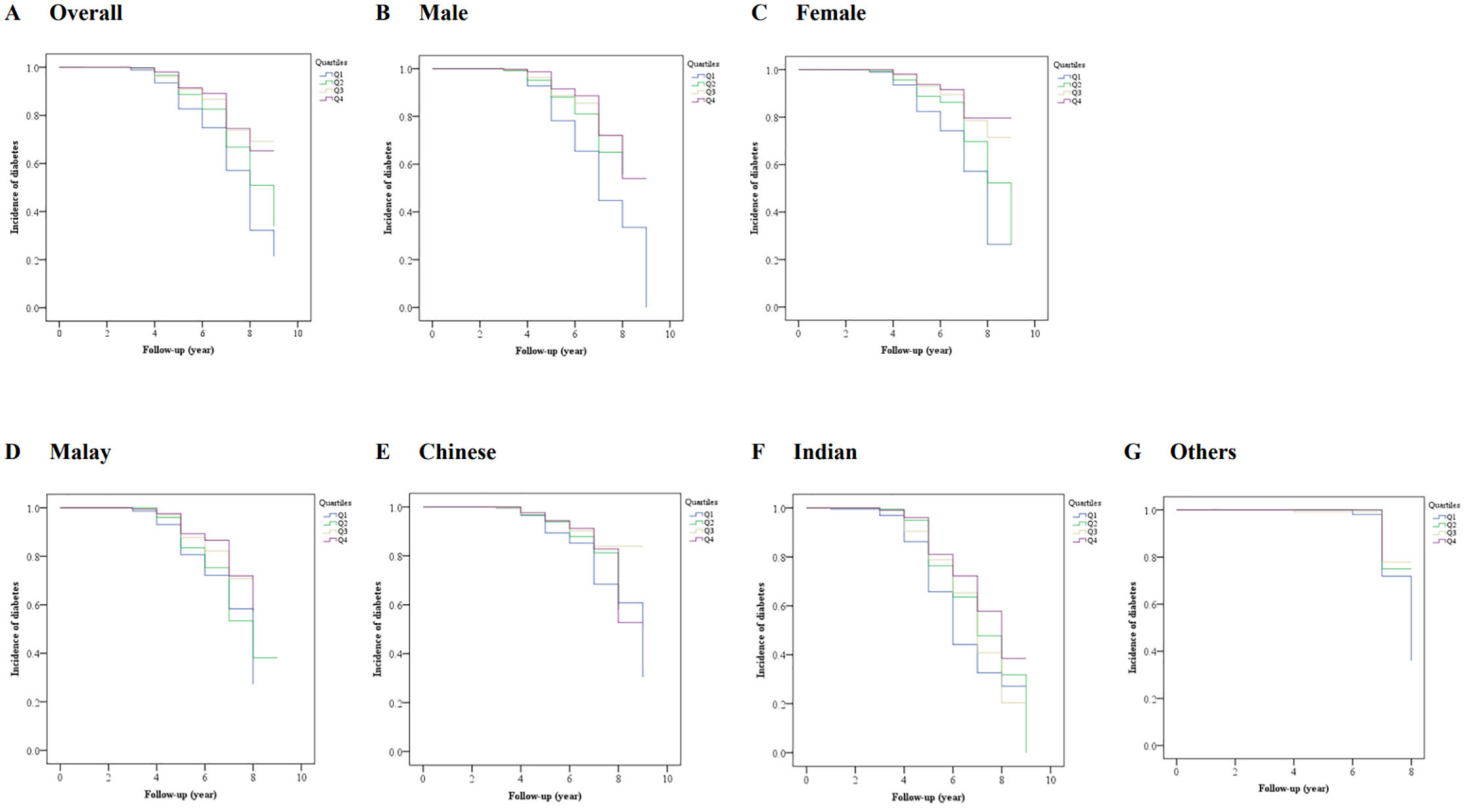
Kaplan–Meier analysis of diabetes risk based on Cre/BW ratio. Kaplan–Meier analysis for incident diabetes (A) as a whole, in (B) male and (C) female, as well as in different ethnicities, including (D) Malay, (E) Chinese, (F) Indian, and (G) other races in Malaysia. The log‐rank test has shown significant (*p* < 0.001) differences for cumulative risk of incident diabetes across Cre/BW ratio quartiles, irrespective of gender and ethnicity.

**FIGURE 5:**
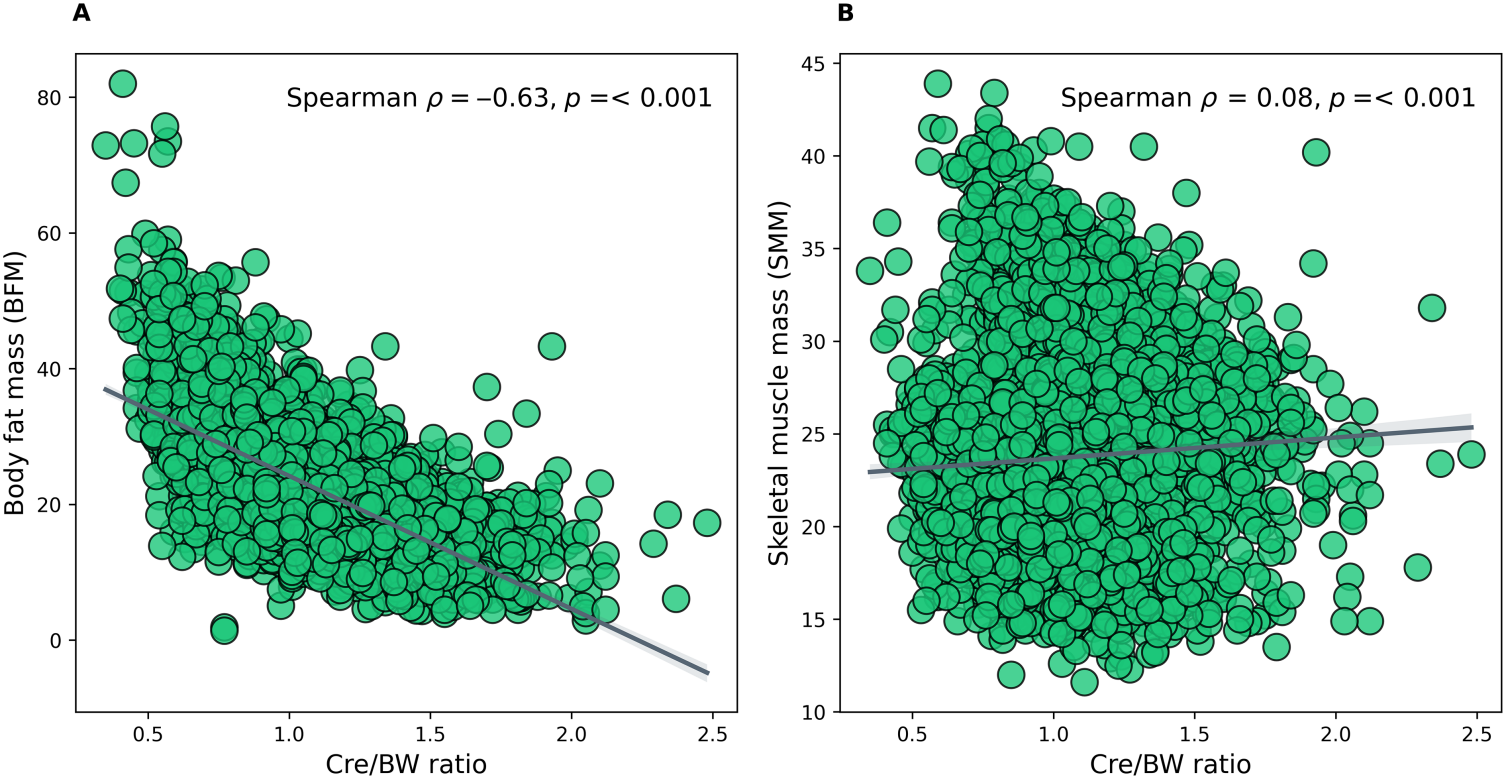
Correlation between Cre/BW ratio and (A) BFM and (B) SMM. *ρ* denotes the Spearman's rank correlation coefficient.

The Kaplan–Meier analysis revealed a significant difference in the risk of having diabetes among Cre/BW ratio quartiles (log‐rank test, *p* < 0.001). In this context, the risk of incident diabetes decreases gradually with increasing Cre/BW ratio quartiles, resulting in the highest risk for those in Q1 (Figure 4A). Significant differences (log‐rank test, *p* < 0.001) were also observed in gender (Figure 4B,C) and ethnicities (Figure 4D–G). When the analysis was stratified based on gender, the females showed a lower HR (HR: 0.441; 95% CI: 0.349–0.559, *p* < 0.001) and longer survival time than males (HR: 0.712; 95% CI: 0.452–1.120, *p* = 0.142) in the final model 3 (Figure 4B,C, and Table S4). On the other hand, when the analysis was classified according to ethnicity, it showed that the Indians have the highest HR (HR: 0.863; 95% CI: 0.519–1.438, *p* = 0.573) of incident diabetes compared to other ethnicities. In addition, this study assessed the correlation between Cre/BW ratio and a specific body composition parameter, either BFM or SMM, estimated using bioelectrical impedance analysis. We found a significant moderate and inversed correlation between Cre/BW ratio and BFM (Spearman's *ρ* = − 0.63, *p* < 0.001; Figure 5A), implying that one with a lower Cre/BW ratio might have higher BFM. In contrast, Cre/BW ratio and SMM showed significant but weak positive correlation (Spearman's *ρ* = 0.08, *p* < 0.001; Figure 5B).

## 4 Discussion

This nested case–control study revealed an inverse association between the Cre/BW ratio and the incidence of diabetes in a multi‐ethnic Malaysian population. This study also suggested Cre/BW ratio as an independent risk factor for incident diabetes after adjusting for age, gender, ethnicity, and other clinical parameters (WHR, HDL‐C, TG, and SBP). The participants in the lowest Cre/BW ratio quartile (Q1) have the highest diabetes risk regardless of gender and ethnicity. Moreover, the Cre/BW ratio appears to be a better predictor of diabetes incidence compared to BMI and waist/height ratio in this study. Based on the Cre/BW ratio's, the male gender and Indian ethnicity have a higher HR of incident diabetes. A significant, moderate, negative correlation was observed between Cre/BW ratio and BFM.

The Cre/BW ratio has been linked to incident diabetes in recent years [5, 6, 7], primarily due to creatinine, a waste product of muscle metabolism. Unlike other indices such as BMI and waist‐to‐height ratio, which are purely anthropometric, the Cre/BW ratio incorporates creatinine levels, providing an indicator of SMM [7]. Serum creatinine levels are relatively stable due to the consistency of total SMM [7] and have been used to measure muscle mass due to their stability [18]. We also shown that Cre/BW ratio is a better predictor comparted to BMI and waist‐to‐height ratio. Studies have shown that low serum creatinine elevates the risk of type 2 diabetes [19, 20]. This is attributed to the lower muscle mass that reduces the capacity of glucose uptake from the blood [21]. Furthermore, muscle mass was associated with insulin resistance, increasing the risk of incident diabetes [22].

The interplay of obesity especially visceral fat, and incident type 2 diabetes has been well established [23, 24]. Visceral fat is associated with ectopic fat [25], which also contributes significantly to the risk of incident type 2 diabetes [26]. The predisposition of fatty tissue also intensifies inflammation and cytokine secretion, altering insulin signaling pathways and eventually causing insulin resistance in obesity [27, 28]. Even without inflammation, excessive and ectopic lipid deposition might form metabolically toxic product, such as ceramide, that leads to insulin resistance [29, 30]. Besides, interactions between visceral fat, obesity, and sarcopenia are significantly associated with metabolic syndrome, a substantial risk of developing type 2 diabetes [31].

On top of that, there is a contrast in the development and severity of type 2 diabetes between the genders due to several differences like biological and psychosocial risk factors and health behaviors [32]. For instance, muscle mass differs between the genders. Men were reported to have a higher SMM and mean serum creatinine value than women [33]. However, the risk of developing diabetes among men is still higher [34]. This is because women are more insulin‐sensitive and capable of insulin secretion and incretin responses than men [34, 35]. In addition, studies have shown that larger amounts of visceral fat in men were linked to a higher prevalence of incident diabetes compared with women [36].

In terms of ethnicity in Malaysia, Indians have the highest prevalence of diabetes, followed by Malays, Chinese, and the indigenous people [4, 37]. Indians are more susceptible to diabetes mellitus than Chinese and Malays due to the naturally higher body fat percentage in Indians than in other ethnicities [38, 39]. Studies have shown that Indian or South Asians may have lower muscle mass and are prone to ectopic fat accumulation [40]. Emerging epidemiological data also indicate that Indians are at higher risk of impaired insulin secretion, adding a further burden to the diabetes incidence among South Asians [40]. Recent studies have shown the association of the Cre/BW ratio in incident diabetes. However, those studies are limited to a certain population in East Asian countries [5, 6, 7]. Thus, this study gives some insight on association between the Cre/CW ratio and incident diabetes in a multi‐ethnic population, which might varies. This study also assessed the correlation of the Cre/BW ratio to a specific body composition, which had not been done in previous studies.

Despite the meaningful findings, there are several limitations in this current study. We did not measure the insulin levels for this study. Thus, we could not associate Cre/BW ratio with insulin resistance. Although a significant, moderate correlation was observed between the Cre/BW ratio and BFM, we could not further distinguish the fat mass as either visceral or ectopic fat. Because of the prospective nature of our cohort study, only 40% participants were successfully followed‐up with a complete data and biospecimens [15]. Thus, to minimize the selection bias, this study was designed as a nested case–control study, which is more efficient and representative than other study designs [41].

## 5 Conclusion

This study demonstrated an inverse relationship between Cre/BW ratio and incident diabetes in a multiethnic population. The association remained significantly inversed regardless of gender and ethnicity. A significant but moderate correlation was also observed between Cre/BW ratio and BFM. Further study is warranted to elucidate the predictive value of the Cre/BW ratio for incident diabetes.

## Author Contributions

Conceptualization: N.A., Y.X.G., and R.J. Data curation: N.A.M.Y., N.A.J., N.I., N.F.H., and M.A.K. Formal analysis: A.N.K. and A.S.K.A. Writing – original draft: N.A. and Y.X.G. Writing – review and editing: R.J. All authors participated in the discussion and interpretation of the results. N.A. and R.J. are the guarantors of this work and, as such, had full access to all the data in the study and take responsibility for the integrity of the data and the accuracy of the data analysis.

## Ethics Statement

This study was approved by the institutional review and ethics board of the Universiti Kebangsaan Malaysia (UKM), Malaysia (Project Code: FF‐205‐2007).

## Conflicts of Interest

The authors declare no conflicts of interest.

## Supporting information


**Data S1.** Supporting Information.

**Table S1.** Baseline characteristics of participants according to the quartiles of Cre/BW ratios, stratified based on gender.

**Table S2.** Baseline characteristics participants according to the quartiles of Cre/BW ratios, stratified based on ethnicities.

**Table S3.** The relationship between Cre/BW ratio and incident diabetes in unadjusted and adjusted proportional hazards models in overall participants.

**Table S4.** The relationship between Cre/BW ratio and incident diabetes in unadjusted and adjusted proportional hazards models, stratified based on gender.

**Table S5.** The relationship between Cre/BW ratio and incident diabetes in unadjusted and adjusted proportional hazards models, stratified based on ethnicity.

**Table S6.** The relationship between BMI, waist/height and incident diabetes in unadjusted and adjusted proportional hazards models in overall participants.

**Table S7.** The relationship between Cre/BW ratio, interaction with ethnicity and incident diabetes in unadjusted and adjusted proportional hazards models in overall participants.

## Data Availability

The data sets generated and analyzed during the current study are not publicly available but are available from the corresponding author on reasonable request.
