# Supplementary material for: Linking Creatinine‐to‐Body Weight Ratio With Diabetes Incidence: A Multiethnic Malaysian Cohort Study
Source: J Diabetes. 2025 Jan 22;17(1):e70039. doi: 10.1111/1753-0407.70039 (PMC11753918; doi:10.1111/1753-0407.70039)
Supplement: Supplementary file 1 — Data S1. Supporting Information. [file JDB-17-e70039-s007.docx]

**Supplementary File 1**

**Hydrogen sulfide and ethylene regulate photosynthesis and sugar metabolism, and tolerance to heat stress in the presence of sulfur in rice**

Ameena Fatima Alvi, Sheen Khan, Nafees A. Khan*

Plant Physiology and Biochemistry Laboratory, Department of Botany, Aligarh Muslim University, Aligarh, 202002, India

*naf9.amu@gmail.com

Determination of oxidative biomarkers

H_2_O_2_

The methodology of Okuda *et al*. (1991) was employed for the estimation of H_2_O_2_ content. 500 mg of fresh leaf tissue was homogenized in ice-cold 200 mM perchloric acid (HClO_4_) and then centrifuged at 1200 x g for 10 minutes. The supernatant obtained was neutralized using 4 M KOH. Further clarification involved centrifugation at 500× g for 3 minutes to remove insoluble potassium perchlorate. For the assessment of H_2_O_2_ levels, a reaction mixture (1.5 ml) was prepared, consisting of 1 mL of the eluate, 80 µL of 3-methyl-2-benzothiazoline hydrazone, 400 µL of 12.5 mM 3-(dimethylamino) benzoic acid in 0.375 M phosphate buffer (pH 6.5), and 20 µL of peroxidase (0.25 unit). The reaction was initiated by adding peroxidase at 25 °C, and the resulting increase in absorbance was measured at 590 nm using a spectrophotometer.

TBARS

Fresh leaf samples weighing 500 mg were crushed in a solution containing 0.25% 2-thiobarbituric acid (TBA) in 10% trichloroacetic acid (TCA) using a mortar and pestle. The mixture underwent heating at 95°C for 30 minutes, followed by rapid cooling in an ice bath and centrifugation at 10,000× g for 10 minutes. To 1 ml of the resulting supernatant, 4.0 ml of 20% TCA containing 5% TBA was added. The absorbance of the supernatant was measured at 532 nm and corrected for non-specific turbidity by subtracting the absorbance at 600 nm. The TBARS content was calculated using an extinction coefficient of 155 mM^−1^ cm^−1^ (Dhindsa *et al.*, 1981).

Antioxidant enzymes

SOD

A 5.0 mL reaction mixture consisting of 5 mM HEPES (pH 7.6), 0.1 mM EDTA, 50 mM Na_2_CO_3_ (pH 10.0), 13 mM methionine, 0.025% (v/v) Triton X-100, 63 µmol NBT, and 1.3 µmol riboflavin was combined with the enzyme-containing extract. This mixture was then exposed to bright light (360 µmol m^−2^ s ^−1^) for 15 minutes. A control sample, not exposed to light, was prepared simultaneously to correct for background absorbance. SOD activity was determined by assessing the enzyme's ability to inhibit NBT reduction by 50%, as measured by the absorbance at 560 nm. One unit of SOD corresponds to the amount of enzyme necessary for this inhibition.

APX

The assay mixture (1.0 mL) consisted of phosphate buffer (50 mM, pH 7.0), 0.1 mM EDTA, 0.5 mM ascorbate, 0.1 mM H_2_O_2_, and enzyme extract. The mixture was observed at 290 nm for 1 minute using a spectrophotometer. A decrease in absorbance was observed immediately upon the addition of H_2_O_2_, indicating the start of the reaction. APX activity was calculated using an extinction coefficient of 2.8 mM^−1^ cm^−1^. One unit of APX corresponds to the amount of enzyme required to decompose one µmol of substrate per minute at 25 °C.

CAT

CAT activity in leaf samples was assessed following the method described by Aebi (1984), which relies on the reduction of H_2_O_2_ during the initiation of the reaction. The reaction mixture comprised 50 mM phosphate buffer (pH 7.0), 15 mM H_2_O_2_, and 100 μL of enzyme extract. The optical density of the reaction mixture was monitored at 240 nm for 2 minutes, with readings taken at 30-second intervals, at 25°C. The same reagents, excluding the enzyme extract, were used as a control.

Cysteine content

The spectrophotometric determination of cysteine (Cys) content in leaves was conducted following the method described by Giatonde (1967). Fresh leaf samples were homogenized in 5% (w/v) ice-cold perchloric acid, with a final volume of 4 ml per gram of plant tissue. The suspension was then centrifuged at 2800 g for 1 hour at 5°C, and the resulting supernatant was filtered through Whatman no.1 paper. A 1 ml aliquot of the filtrate was treated with acid ninhydrin reagent, and the absorbance was measured at 580 nm. The quantity of Cysteine was determined by reference to a calibration curve generated under similar conditions using standard Cysteine.

GSH content

To assess reduced glutathione (GSH), an enzyme recycling method was employed, wherein GSH was first oxidized by 5,5-dithiobis-2-nitrobenzoic acid (DTNB) and then reduced back by NADPH in the presence of glutathione reductase (GR). For the specific determination of oxidized glutathione (GSSG), GSH was masked by derivatization with 2-vinylpyridine. Fresh leaf tissues (500 mg) were pulverized in liquid nitrogen using a mortar and pestle and suspended in 2 mL of 5% (w/v) sulfosalicylic acid. Following centrifugation at 12,000×g for 10 minutes, a 300 μL aliquot of the supernatant was taken and neutralized by adding 18 μL of 7.5 M triethanolamine. To determine the concentrations of both GSH and GSSG, a 150 μL sample was used. Another sample underwent pretreatment with 3 μL of 2-vinylpyridine for 60 minutes at 20°C to derivatize and mask GSH, allowing for the subsequent determination of GSSG alone. In each case, 50 μL aliquots of the samples were mixed with 700 μL of 0.3 mM NADPH, 100 μL of DTNB, and 150 μL of buffer containing 125 mM sodium phosphate and 6.3 mM EDTA at pH 6.5. Subsequently, a 10 μL aliquot of GR (5 U mL^-1^) was added, and the change in absorbance at 412 nm was monitored at 30°C. Standard curves were prepared for GSH and GSSG, covering ranges of 5-55 nmol and 1-5 nmol, respectively.

Quantitative Real-Time Reverse Transcription–Polymerase Chain Reaction Analysis

Total RNA was extracted from 100 mg of fresh leaves using a Trizol reagent according to the manufacturer's instructions. Impurities in the RNA were removed using an RNase-free DNase kit, and the yields were quantified using a microplate reader. RNA integrity was assessed via formaldehyde gel electrophoresis by separating equal volumes of each sample. First-strand complementary DNA was synthesized using a Verso cDNA synthesis kit on DNA-free total RNA. For gene expression analysis, specific primers were designed and quantitative real-time polymerase chain reaction (qRT-PCR) was performed using Maxima SYBR Green/ROX qPCR Master Mix on a Light Cycler. Biological and technical replicates were included in qRT-PCR reactions, and the target gene expression was normalized to actin expression using the 2^(-ΔΔCt) method (Livak and Schimittgen 2001)

**Table1: Primer references for RT-PCR**

| **S.No.** | **Gene** | **Forward primer** | **Reverse primer** | **Gene ID** |
| --- | --- | --- | --- | --- |
| 1. | *Ribulose-1,5-bisphosphatase carboxylase/oxygenase large subunit* (*rbcL*) | AAGGCCCGCCTCATGGTATC | GTCCACCGGCTAGACACTCA | 476751 |
| 2. | *Ribulose-1,5-bisphosphatase carboxylase/oxygenase small subunit* (*rbcS*) | AAGAAGGCCTACCCCGATGC | ACCAGCGCGATGCTTGATCT | 38374127 |
| 3. | *Sucrose transport 1* | TCATCCCTCAGGTGGTCATCG | CTTGGAGATCTTGGGCAGCAG | 100027 |
| 4. | *Sucrose synthase 2* | TTCAGCAGGAGAAGCCGTCAGC | CCGGCGTTTATTTGAGGCAAGC | 072074 |
| **Reference gene primer sequences used for quantitative RT-PCR** | | | | |
| 1. | *Ubiquitin* | AGAACAAGCTGGTTTGCCGC | CGACGGAGCTTTCCAGTGGT | 1443098341 |

**References**

**Aebi H.** 1984. Catalase in vitro. In Packer L ed., Methods in enzymology. New York: Academic press. Inc., **105**,121-126.

**Dhindsa, R. S., Plumb-Dhindsa P, Thorpe TA.** 1981. Leaf senescence: correlated with increased levels of membrane permeability and lipid peroxidation, and decreased levels of superoxide dismutase and catalase. Journal of Experimental botany**32**, 93-101.

**Gaitonde MK.** 1967. A spectrophotometric method for the direct determination of cysteine in the presence of other naturally occurring amino acids. Biochemical Journal **104**, 627.

**Livak KJ, Schmittgen TD.** 2001. Analysis of relative gene expression data using real-time quantitative PCR and the 2− ΔΔCT method. Methods **25**, 402-408

**Okuda T, Matsuda Y, Yamanaka A, Sagisaka S.** 1991. Abrupt increase in the level of hydrogen peroxide in leaves of winter wheat is caused by cold treatment. Plant physiology **97**, 1265-1267.
