# Supplementary material for: Linking Creatinine‐to‐Body Weight Ratio With Diabetes Incidence: A Multiethnic Malaysian Cohort Study
Source: J Diabetes. 2025 Jan 22;17(1):e70039. doi: 10.1111/1753-0407.70039 (PMC11753918; doi:10.1111/1753-0407.70039)
Supplement: Supplementary file 2 — Table S1. Baseline characteristics of participants according to the quartiles of Cre/BW ratios, stratified based on gender. [file JDB-17-e70039-s002.docx]

**Supplementary Table S1** Baseline characteristics of participants according to the quartiles of Cre/BW ratios, stratified based on gender

| **Cre/BW** | **Overall male** | **Male, by Cre/BW quartiles** | | | | |
| --- | --- | --- | --- | --- | --- | --- |
|  |  | **Q1 (< 1.0092)** | **Q2 (1.0092 ≤ to < 1.1872)** | **Q3 (1.1872 ≤ to < 1.3670)** | **Q4 (≥ 1.3670)** | ***P*-value** |
| Count (*n*) | 1850 | 459 | 478 | 440 | 473 |  |
| Age (years) | 49 ± 7.81 | 47.34 ± 7.65 | 48.35 ± 7.42 | 48.66 ± 7.69 | 51.59 ± 7.84 | < 0.001* |
| Height (cm) | 167.44 ± 6.35 | 170.19 ± 6.4 | 168.38 ± 5.7 | 166.51 ± 6.18 | 164.7 ± 5.78 | < 0.001* |
| Weight (kg) | 72.51 ± 12.95 | 85.13 ± 12.99 | 73.6 ± 8.85 | 68.65 ± 8.98 | 62.75 ± 8.6 | < 0.001* |
| WHR | 0.9 ± 0.06 | 0.93 ± 0.06 | 0.9 ± 0.05 | 0.89 ± 0.06 | 0.88 ± 0.06 | < 0.001* |
| HDL Cholesterol (mmol/L) | 1.24 ± 0.31 | 1.2 ± 0.28 | 1.24 ± 0.33 | 1.25 ± 0.32 | 1.29 ± 0.32 | < 0.001* |
| LDL Cholesterol (mmol/L) | 3.65 ± 0.94 | 3.56 ± 0.89 | 3.68 ± 0.94 | 3.7 ± 0.96 | 3.65 ± 0.99 | 0.111 |
| Triglycerides (mmol/L) | 1.69 ± 1.13 | 1.76 ± 1.18 | 1.76 ± 1.28 | 1.72 ± 1.06 | 1.54 ± 0.95 | 0.008* |
| Systolic Blood Pressure (mmHg) | 130.13 ± 17.68 | 132.36 ± 16.8 | 129.39 ± 16.62 | 128.56 ± 17.12 | 130.15 ± 19.77 | 0.009* |
| Diastolic Blood Pressure (mmHg) | 78.31 ± 11.2 | 80.86 ± 11.36 | 77.89 ± 10.45 | 77.36 ± 11.39 | 77.14 ± 11.24 | < 0.001* |
| HbA1C (%) | 5.52 ± 0.11 | 5.53 ± 0.12 | 5.52 ± 0.1 | 5.51 ± 0.13 | 5.52 ± 0.09 | 0.235 |
| TC (mmol/L) | 5.64 ± 1.06 | 5.55 ± 1 | 5.69 ± 1.07 | 5.69 ± 1.04 | 5.63 ± 1.11 | 0.126 |
| FPG (mmol/L) | 5.37 ± 0.39 | 5.39 ± 0.36 | 5.39 ± 0.4 | 5.35 ± 0.37 | 5.33 ± 0.41 | 0.033* |
| Waist Circumference (cm) | 88.23 ± 10.7 | 97.31 ± 10.49 | 89.09 ± 8 | 85.67 ± 8.15 | 80.92 ± 8.64 | < 0.001* |
| BMI (kg/m²) | 25.8 ± 4.07 | 29.31 ± 4.04 | 25.98 ± 3.13 | 24.78 ± 3.14 | 23.16 ± 3.16 | < 0.001* |
| Cre (umol/L) | 85.02 ± 14.46 | 74 ± 9.25 | 80.58 ± 9.52 | 87.5 ± 11.29 | 97.88 ± 14.71 | < 0.001* |
| **Ethnicity** |  |  |  |  |  | < 0.001* |
| Malay | 676 (36.5) | 149 (22) | 173 (25.6) | 169 (25) | 185 (27.4) |  |
| Chinese | 620 (33.5) | 165 (26.6) | 182 (29.4) | 135 (21.8) | 138 (22.3) |  |
| Indian | 369 (19.9) | 109 (29.5) | 95 (25.7) | 88 (23.8) | 77 (20.9) |  |
| Others | 185 (10) | 36 (19.5) | 28 (15.1) | 48 (25.9) | 73 (39.5) |  |
| **Abdominal Obesity** |  |  |  |  |  | < 0.001* |
| Yes | 185 (10) | 145 (31.6) | 27 (5.6) | 11 (2.5) | 2 (0.4) |  |
| No | 1665 (90) | 314 (68.4) | 451 (94.4) | 429 (97.5) | 471 (99.6) |  |
| **Obesity (kg/m^2^)** |  |  |  |  |  | < 0.001* |
| Underweight  (< 18.5) | 40 (2.2) | 0 (0) | 1 (2.5) | 6 (15) | 33 (82.5) |  |
| Normal  (18.5 - 22.9) | 376 (20.3) | 14 (3.7) | 68 (18.1) | 116 (30.9) | 178 (47.3) |  |
| Overweight  (23.0 - 27.4) | 858 (46.4) | 142 (16.6) | 258 (30.1) | 240 (28) | 218 (25.4) |  |
| Obese  (> 27.5) | 576 (31.1) | 303 (52.6) | 151 (26.2) | 78 (13.5) | 44 (7.6) |  |
| **Smoking Status** |  |  |  |  |  | 0.737 |
| Yes | 904 (48.9) | 226 (25) | 227 (25.1) | 224 (24.8) | 227 (25.1) |  |
| No | 946 (51.1) | 233 (24.6) | 251 (26.5) | 216 (22.8) | 246 (26) |  |
| **Drinking Status** |  |  |  |  |  | 0.133 |
| Yes | 173 (9.4) | 48 (27.7) | 42 (24.3) | 35 (20.2) | 48 (27.7) |  |
| No | 1332 (72) | 276 (20.7) | 359 (27) | 338 (25.4) | 359 (27) |  |
| Unknown | 345 (18.6) | 135 (39.1) | 77 (22.3) | 67 (19.4) | 66 (19.1) |  |
| **Developed Incident T2DM** | |  |  |  |  | < 0.001* |
| Yes | 350 (18.9) | 138 (39.4) | 78 (22.3) | 67 (19.1) | 67 (19.1) |  |
| No | 1500 (81.1) | 321 (21.4) | 400 (26.7) | 373 (24.9) | 406 (27.1) |  |
|  | **Overall female** | **Female, by Cre/BW quartiles** | | | | |
|  |  | **Q1 (< 0.7990)** | **Q2 ( 0.7990 ≤ to < 0.9325)** | **Q3 (0.9325 ≤ to 1.0926)** | **Q4 (< 1.0926)** | ***P*-value** |
| Count (*n*) | 2650 | 663 | 615 | 704 | 668 |  |
| Age (years) | 47.48 ± 7.37 | 46.31 ± 6.95 | 46.76 ± 6.92 | 47.99 ± 7.44 | 48.78 ± 7.84 | < 0.001* |
| Height (cm) | 155.28 ± 5.69 | 156.7 ± 5.48 | 155.98 ± 5.7 | 154.89 ± 5.56 | 153.62 ± 5.55 | < 0.001* |
| Weight (kg) | 61.79 ± 12.28 | 73.3 ± 13.36 | 63.23 ± 8.6 | 58.4 ± 7.76 | 52.61 ± 7.73 | < 0.001* |
| WHR | 0.82 ± 0.07 | 0.84 ± 0.07 | 0.82 ± 0.07 | 0.81 ± 0.07 | 0.8 ± 0.07 | < 0.001* |
| HDL Cholesterol (mmol/L) | 1.57 ± 0.41 | 1.44 ± 0.34 | 1.57 ± 0.42 | 1.59 ± 0.41 | 1.67 ± 0.43 | < 0.001* |
| LDL Cholesterol (mmol/L) | 3.46 ± 0.93 | 3.41 ± 0.95 | 3.48 ± 0.98 | 3.49 ± 0.88 | 3.46 ± 0.91 | 0.382 |
| Triglycerides (mmol/L) | 1.25 ± 0.65 | 1.32 ± 0.7 | 1.24 ± 0.62 | 1.24 ± 0.66 | 1.2 ± 0.61 | 0.013* |
| Systolic Blood Pressure (mmHg) | 126.43 ± 19.08 | 128.29 ± 18.86 | 125.07 ± 18.37 | 126.17 ± 18.79 | 126.11 ± 20.14 | 0.021* |
| Diastolic Blood Pressure (mmHg) | 75.57 ± 10.8 | 77.7 ± 11.39 | 75.14 ± 10.62 | 75.21 ± 10.34 | 74.23 ± 10.53 | < 0.001* |
| HbA1C (%) | 5.52 ± 0.11 | 5.52 ± 0.11 | 5.53 ± 0.13 | 5.51 ± 0.11 | 5.52 ± 0.08 | 0.025* |
| TC (mmol/L) | 5.56 ± 1.03 | 5.43 ± 1.05 | 5.58 ± 1.05 | 5.6 ± 0.99 | 5.62 ± 1 | 0.002* |
| FPG (mmol/L) | 5.21 ± 0.41 | 5.26 ± 0.42 | 5.21 ± 0.4 | 5.19 ± 0.39 | 5.19 ± 0.43 | 0.005* |
| Waist Circumference (cm) | 80.91 ± 11.26 | 89.84 ± 11.59 | 82.21 ± 8.98 | 78.23 ± 8.52 | 73.66 ± 8.84 | < 0.001* |
| BMI (kg/m²) | 25.6 ± 4.83 | 29.78 ± 5.22 | 26.05 ± 3.75 | 24.39 ± 3.36 | 22.34 ± 3.34 | < 0.001* |
| Cre (umol/L) | 57.59 ± 10.2 | 50.24 ± 8.34 | 54.87 ± 7.35 | 59.03 ± 7.81 | 65.87 ± 9.94 | < 0.001* |
| **Ethnicity** |  |  |  |  |  | < 0.001* |
| Malay | 831 (31.4) | 246 (29.6) | 191 (23) | 202 (24.3) | 192 (23.1) |  |
| Chinese | 1123 (42.4) | 238 (21.2) | 244 (21.7) | 333 (29.7) | 308 (27.4) |  |
| Indian | 431 (16.3) | 123 (28.5) | 127 (29.5) | 97 (22.5) | 84 (19.5) |  |
| Others | 265 (10) | 56 (21.1) | 53 (20) | 72 (27.2) | 84 (31.7) |  |
| **Abdominal Obesity** |  |  |  |  |  | < 0.001* |
| Yes | 635 (24) | 352 (53.1) | 158 (25.7) | 83 (11.8) | 42 (6.3) |  |
| No | 2015 (76) | 311 (46.9) | 457 (74.3) | 621 (88.2) | 626 (93.7) |  |
| **Obesity (kg/m^2^)** |  |  |  |  |  | < 0.001* |
| Underweight  (< 18.5) | 96 (3.6) | 2 (2.1) | 4 (4.2) | 12 (12.5) | 78 (81.3) |  |
| Normal  (18.5 - 22.9) | 741 (28) | 50 (6.7) | 135 (18.2) | 239 (32.3) | 317 (42.8) |  |
| Overweight  (23.0 - 27.4) | 972 (36.7) | 180 (18.5) | 257 (26.4) | 320 (32.9) | 215 (22.1) |  |
| Obese  (> 27.5) | 841 (31.7) | 431 (51.2) | 219 (26) | 133 (15.8) | 58 (6.9) |  |
| **Smoking Status** |  |  |  |  |  | 0.007* |
| Yes | 78 (2.9) | 17 (21.8) | 10 (12.8) | 19 (24.4) | 32 (41) |  |
| No | 2572 (97.1) | 646 (25.1) | 605 (23.5) | 685 (26.6) | 636 (24.7) |  |
| **Drinking Status** |  |  |  |  |  | 0.458 |
| Yes | 34 (1.3) | 9 (26.5) | 4 (11.8) | 11 (32.4) | 10 (29.4) |  |
| No | 2224 (83.9) | 488 (21.9) | 521 (23.4) | 621 (27.9) | 594 (26.7) |  |
| Unknown | 392 (14.8) | 166 (42.3) | 90 (23) | 72 (18.4) | 64 (16.3) |  |
| **Developed Incident T2DM** | |  |  |  |  | < 0.001* |
| Yes | 400 (15.1) | 169 (42.3) | 93 (23.3) | 74 (18.5) | 64 (16) |  |
| No | 2250 (84.9) | 494 (22) | 522 (23.2) | 630 (28) | 604 (26.8) |  |

Data are given as mean ± standard deviation (SD), or frequency (percentage), as appropriate. **P* < 0.05. BMI, body mass index; Cre/BW, creatinine-to-body weight; HbA1c, haemoglobin A1c; HDL-C, high-density lipoprotein cholesterol; LDL-C, low-density lipoprotein cholesterol; T2DM, type 2 diabetes mellitus.
