# Supplementary material for: Linking Creatinine‐to‐Body Weight Ratio With Diabetes Incidence: A Multiethnic Malaysian Cohort Study
Source: J Diabetes. 2025 Jan 22;17(1):e70039. doi: 10.1111/1753-0407.70039 (PMC11753918; doi:10.1111/1753-0407.70039)
Supplement: Supplementary file 3 — Table S2. Baseline characteristics participants according to the quartiles of Cre/BW ratios, stratified based on ethnicities. [file JDB-17-e70039-s003.docx]

**Supplementary Table S2** Baseline characteristics participants according to the quartiles of Cre/BW ratios, stratified based on ethnicities

| **Cre/BW** | **Overall Malay** | **Malay, by Cre/BW quartiles** | | | | |
| --- | --- | --- | --- | --- | --- | --- |
|  |  | **Q1 (< 0.8542)** | **Q2 (0.8542 ≤ to < 1.031)** | **Q3 (1.031 ≤ to < 1.2650)** | **Q4 ≥ 1.2650)** | ***P*-value** |
| Count (*n*) | 1507 | 367 | 361 | 435 | 344 |  |
| Age (years) | 47.4 ± 7.33 | 45.81 ± 6.77 | 46.61 ± 6.82 | 47.69 ± 7.47 | 49.53 ± 7.73 | < 0.001* |
| Height (cm) | 159.9 ± 8.35 | 157.05 ± 7.35 | 159.26 ± 8.64 | 160.82 ± 9 | 162.47 ± 7.14 | < 0.001* |
| Weight (kg) | 68.2 ± 13.54 | 75.47 ± 16.52 | 68.74 ± 12.01 | 65.48 ± 11.35 | 63.32 ± 10.45 | < 0.001* |
| WHR | 0.85 ± 0.08 | 0.85 ± 0.08 | 0.85 ± 0.08 | 0.86 ± 0.08 | 0.86 ± 0.07 | 0.038* |
| HDL Cholesterol (mmol/L) | 1.4 ± 0.37 | 1.44 ± 0.33 | 1.44 ± 0.38 | 1.38 ± 0.38 | 1.37 ± 0.39 | 0.007* |
| LDL Cholesterol (mmol/L) | 3.69 ± 0.97 | 3.56 ± 0.98 | 3.64 ± 0.94 | 3.77 ± 0.97 | 3.79 ± 0.98 | 0.003* |
| Triglycerides (mmol/L) | 1.43 ± 0.85 | 1.34 ± 0.74 | 1.4 ± 0.8 | 1.47 ± 0.85 | 1.51 ± 1 | 0.036* |
| Systolic Blood Pressure (mmHg) | 127.91 ± 18.48 | 128.78 ± 18.87 | 127.88 ± 18.62 | 127.28 ± 18.33 | 127.8 ± 18.15 | 0.725 |
| Diastolic Blood Pressure (mmHg) | 76.95 ± 11.23 | 78.32 ± 11.89 | 77.27 ± 11.17 | 76 ± 10.77 | 76.36 ± 11.04 | 0.020* |
| HbA1C (%) | 5.52 ± 0.09 | 5.53 ± 0.09 | 5.52 ± 0.08 | 5.52 ± 0.09 | 5.52 ± 0.09 | 0.567 |
| TC (mmol/L) | 5.72 ± 1.07 | 5.6 ± 1.09 | 5.69 ± 1.04 | 5.79 ± 1.07 | 5.81 ± 1.08 | 0.032* |
| FPG (mmol/L) | 5.26 ± 0.41 | 5.23 ± 0.41 | 5.26 ± 0.4 | 5.29 ± 0.41 | 5.27 ± 0.41 | 0.172 |
| Waist Circumference (cm) | 84.97 ± 11.26 | 90.37 ± 12.67 | 85.05 ± 10.43 | 83.33 ± 10.11 | 81.22 ± 9.67 | < 0.001* |
| BMI (kg/m²) | 26.59 ± 4.53 | 30.33 ± 5.28 | 26.97 ± 3.26 | 25.23 ± 3.31 | 23.93 ± 3.32 | < 0.001* |
| Cre (umol/L) | 71.47 ± 19.4 | 54.17 ± 10.91 | 64.9 ± 11.93 | 74.25 ± 13.23 | 93.31 ± 17.32 | < 0.001* |
| **Gender** |  |  |  |  |  | < 0.001* |
| Male | 676 (44.9) | 47 (7) | 120 (17.8) | 232 (34.3) | 277 (41) |  |
| Female | 831 (55.1) | 320 (38.5) | 241 (29) | 203 (24.4) | 67 (8.1) |  |
| **Abdominal Obesity** |  |  |  |  |  | < 0.001* |
| Yes | 289 (19.2) | 179 (48.8) | 66 (18.3) | 37 (8.5) | 7 (2) |  |
| No | 1218 (80.8) | 188 (51.2) | 295 (81.7) | 398 (91.5) | 337 (98) |  |
| **Obesity (kg/m^2^)** |  |  |  |  |  | < 0.001* |
| Underweight  (< 18.5) | 27 (1.8) | 1 (3.7) | 0 (0) | 7 (25.9) | 19 (70.4) |  |
| Normal  (18.5 - 22.9) | 268 (17.8) | 24 (9) | 43 (16) | 97 (36.2) | 104 (38.8) |  |
| Overweight  (23.0 - 27.4) | 637 (42.3) | 84 (13.2) | 160 (25.1) | 221 (34.7) | 172 (27) |  |
| Obese  (> 27.5) | 575 (38.2) | 258 (44.9) | 158 (27.5) | 110 (19.1) | 49 (8.5) |  |
| **Smoking Status** |  |  |  |  |  | < 0.001* |
| Yes | 419 (27.8) | 27 (6.4) | 87 (20.8) | 145 (34.6) | 160 (38.2) |  |
| No | 1088 (72.2) | 340 (31.3) | 274 (25.2) | 290 (26.7) | 184 (16.9) |  |
| **Drinking Status** |  |  |  |  |  | 0.238 |
| Yes | 17 (1.1) | 1 (5.9) | 4 (23.5) | 5 (29.4) | 7 (41.2) |  |
| No | 1226 (81.4) | 279 (22.8) | 283 (23.1) | 372 (30.3) | 292 (23.8) |  |
| Unknown | 264 (17.5) | 87 (33) | 74 (28) | 58 (22) | 45 (17) |  |
| **Developed Incident T2DM** | |  |  |  |  | < 0.001* |
| Yes | 266 (17.7) | 88 (24) | 75 (20.8) | 58 (13.3) | 45 (13.1) |  |
| No | 1241 (82.3) | 279 (76) | 286 (79.2) | 377 (86.7) | 299 (86.9) |  |
|  | **Overall Chinese** | **Chinese, by Cre/BW quartiles** | | | | |
|  |  | **Q1 < 0.8707)** | **Q2 (0.8707 ≤ to < 1.0256)** | **Q3 (1.0256 ≤ to < 1.2047)** | **Q4 (≥ 1.2047)** | ***P*-value** |
| Count (*n*) | 1743 | 414 | 443 | 447 | 439 |  |
| Age (years) | 48.62 ± 7.82 | 47.06 ± 7.45 | 48.04 ± 7.72 | 49.06 ± 7.49 | 50.24 ± 8.24 | < 0.001* |
| Height (cm) | 161.17 ± 8.22 | 161.16 ± 8.12 | 160.56 ± 7.71 | 160.87 ± 8.82 | 162.09 ± 8.14 | 0.036* |
| Weight (kg) | 62.66 ± 12.78 | 70.22 ± 14.52 | 62.49 ± 11.12 | 60.49 ± 11.17 | 57.9 ± 10.85 | < 0.001 |
| WHR | 0.84 ± 0.08 | 0.85 ± 0.08 | 0.83 ± 0.07 | 0.83 ± 0.08 | 0.84 ± 0.08 | 0.003* |
| HDL Cholesterol (mmol/L) | 1.56 ± 0.43 | 1.52 ± 0.42 | 1.61 ± 0.43 | 1.56 ± 0.45 | 1.55 ± 0.43 | 0.033* |
| LDL Cholesterol (mmol/L) | 3.41 ± 0.91 | 3.3 ± 0.91 | 3.4 ± 0.95 | 3.47 ± 0.81 | 3.46 ± 0.96 | 0.030* |
| Triglycerides (mmol/L) | 1.4 ± 1 | 1.42 ± 1.12 | 1.38 ± 0.93 | 1.44 ± 1.15 | 1.37 ± 0.77 | 0.697 |
| Systolic Blood Pressure (mmHg) | 127.32 ± 18.56 | 127.61 ± 18.62 | 126.34 ± 17.81 | 127.96 ± 17.54 | 127.38 ± 20.21 | 0.599 |
| Diastolic Blood Pressure (mmHg) | 75.48 ± 10.89 | 76.06 ± 11 | 75.36 ± 10.9 | 75.5 ± 10.23 | 75.03 ± 11.43 | 0.579 |
| HbA1C (%) | 5.51 ± 0.14 | 5.52 ± 0.16 | 5.51 ± 0.15 | 5.51 ± 0.13 | 5.51 ± 0.13 | 0.417 |
| TC (mmol/L) | 5.56 ± 1.01 | 5.41 ± 1.01 | 5.6 ± 1.05 | 5.63 ± 0.94 | 5.59 ± 1.04 | 0.007* |
| FPG (mmol/L) | 5.27 ± 0.39 | 5.27 ± 0.4 | 5.23 ± 0.36 | 5.27 ± 0.42 | 5.3 ± 0.39 | 0.096 |
| Waist Circumference (cm) | 80.66 ± 10.85 | 86.88 ± 11.7 | 80.4 ± 9.53 | 78.99 ± 9.73 | 76.78 ± 9.81 | < 0.001* |
| BMI (kg/m²) | 24.02 ± 3.93 | 26.87 ± 4.34 | 24.14 ± 3.25 | 23.26 ± 3.07 | 21.97 ± 3.27 | < 0.001* |
| Cre (umol/L) | 64.64 ± 16.23 | 52.45 ± 11.38 | 59.42 ± 10.85 | 66.77 ± 12.5 | 79.21 ± 16.21 | < 0.001* |
| **Gender** |  |  |  |  |  | < 0.001* |
| Male | 620 (35.6) | 76 (12.3) | 107 (17.3) | 173 (27.9) | 264 (42.6) |  |
| Female | 1123 (64.4) | 338 (30.1) | 336 (29.9) | 274 (24.4) | 175 (15.6) |  |
| **Abdominal Obesity** |  |  |  |  |  | < 0.001* |
| Yes | 195 (11.2) | 131 (31.6) | 36 (8.1) | 14 (3.1) | 14 (3.2) |  |
| No | 1548 (88.8) | 283 (68.4) | 407 (91.9) | 433 (96.9) | 425 (96.8) |  |
| **Obesity (kg/m^2^)** |  |  |  |  |  | < 0.001* |
| Underweight  (< 18.5) | 90 (5.2) | 2 (2.2) | 10 (11.1) | 20 (22.2) | 58 (64.4) |  |
| Normal  (18.5 - 22.9) | 647 (37.1) | 72 (11.1) | 164 (25.3) | 190 (29.4) | 221 (34.2) |  |
| Overweight  (23.0 - 27.4) | 681 (39.1) | 167 (24.5) | 193 (28.3) | 190 (27.9) | 131 (19.2) |  |
| Obese  (> 27.5) | 325 (18.6) | 173 (53.2) | 76 (23.4) | 47 (14.5) | 29 (8.9) |  |
| **Smoking Status** |  |  |  |  |  | < 0.001* |
| Yes | 326 (18.7) | 57 (17.5) | 57 (17.5) | 82 (25.2) | 130 (39.9) |  |
| No | 1417 (81.3) | 357 (25.2) | 386 (27.2) | 365 (25.8) | 309 (21.8) |  |
| **Drinking Status** |  |  |  |  |  | 0.151 |
| Yes | 122 (7) | 20 (16.4) | 30 (24.6) | 32 (26.2) | 40 (32.8) |  |
| No | 1451 (83.2) | 340 (23.4) | 371 (25.6) | 380 (26.2) | 360 (24.8) |  |
| Unknown | 170 (9.8) | 54 (31.8) | 42 (24.7) | 35 (20.6) | 39 (22.9) |  |
| **Developed Incident T2DM** | |  |  |  |  | 0.024* |
| Yes | 178 (10.2) | 58 (32.6) | 44 (24.7) | 36 (20.2) | 40 (22.5) |  |
| No | 1565 (89.8) | 356 (22.7) | 399 (25.5) | 411 (26.3) | 399 (25.5) |  |
|  | **Overall Indian** | **Indian, by Cre/BW quartiles** | | | | |
|  |  | **Q1 < 0.8380)** | **Q2 (0.8380 ≤ to < 0.9953)** | **Q3 (0.9953 ≤ to < 1.1953)** | **Q4 ≥ 1.1953)** | ***P*-value** |
| Count (*n*) | 800 | 194 | 191 | 201 | 214 |  |
| Age (years) | 47.89 ± 7.49 | 46.47 ± 7.04 | 47.66 ± 7.26 | 47.96 ± 7.36 | 49.33 ± 7.99 | 0.002* |
| Height (cm) | 161.11 ± 9 | 159.3 ± 8.51 | 159.41 ± 8.75 | 162.06 ± 9.46 | 163.38 ± 8.63 | < 0.001* |
| Weight (kg) | 70.34 ± 13.99 | 79.44 ± 14.81 | 71.47 ± 12.79 | 67.42 ± 12.05 | 63.84 ± 11.22 | < 0.001* |
| WHR | 0.88 ± 0.08 | 0.88 ± 0.09 | 0.87 ± 0.09 | 0.88 ± 0.08 | 0.89 ± 0.08 | 0.328 |
| HDL Cholesterol (mmol/L) | 1.25 ± 0.32 | 1.26 ± 0.33 | 1.28 ± 0.33 | 1.23 ± 0.32 | 1.24 ± 0.32 | 0.372 |
| LDL Cholesterol (mmol/L) | 3.6 ± 0.91 | 3.51 ± 0.87 | 3.57 ± 0.97 | 3.66 ± 0.87 | 3.66 ± 0.9 | 0.279 |
| Triglycerides (mmol/L) | 1.47 ± 0.75 | 1.31 ± 0.52 | 1.46 ± 0.69 | 1.53 ± 0.79 | 1.58 ± 0.92 | 0.003* |
| Systolic Blood Pressure (mmHg) | 126.24 ± 18.03 | 127.36 ± 18.13 | 125.8 ± 17.47 | 125.22 ± 18.1 | 126.58 ± 18.42 | 0.666 |
| Diastolic Blood Pressure (mmHg) | 77.72 ± 10.89 | 79.09 ± 10.26 | 78 ± 10.79 | 77.12 ± 10.88 | 76.8 ± 11.46 | 0.149 |
| HbA1C (%) | 5.53 ± 0.09 | 5.53 ± 0.09 | 5.53 ± 0.14 | 5.53 ± 0.08 | 5.52 ± 0.04 | 0.764 |
| TC (mmol/L) | 5.5 ± 1.01 | 5.36 ± 0.99 | 5.5 ± 1.08 | 5.55 ± 0.98 | 5.6 ± 1 | 0.107 |
| FPG (mmol/L) | 5.29 ± 0.42 | 5.29 ± 0.42 | 5.28 ± 0.43 | 5.29 ± 0.42 | 5.3 ± 0.42 | 0.953 |
| Waist Circumference (cm) | 88.88 ± 11.96 | 96.22 ± 12.47 | 90.23 ± 11.21 | 86.52 ± 10.14 | 83.26 ± 9.98 | < 0.001* |
| BMI (kg/m²) | 27.03 ± 4.71 | 31.14 ± 4.74 | 28.02 ± 3.78 | 25.57 ± 3.45 | 23.81 ± 3.22 | < 0.001* |
| Cre (umol/L) | 71.49 ± 18.1 | 57.08 ± 10.53 | 65.04 ± 11.96 | 73.36 ± 13.68 | 88.55 ± 17.41 | < 0.001* |
| **Gender** |  |  |  |  |  | < 0.001* |
| Male | 369 (46.1) | 38 (10.3) | 59 (16) | 109 (29.5) | 163 (44.2) |  |
| Female | 431 (53.9) | 156 (36.2) | 132 (30.6) | 92 (21.3) | 51 (11.8) |  |
| **Abdominal Obesity** |  |  |  |  |  | < 0.001* |
| Yes | 237 (29.6) | 131 (67.5) | 78 (40.8) | 21 (10.4) | 7 (3.3) |  |
| No | 563 (70.4) | 63 (32.5) | 113 (59.2) | 180 (89.6) | 207 (96.7) |  |
| **Obesity (kg/m^2^)** |  |  |  |  |  | < 0.001* |
| Underweight  (< 18.5) | 13 (1.6) | 0 (0) | 0 (0) | 3 (23.1) | 10 (76.9) |  |
| Normal  (18.5 - 22.9) | 126 (15.8) | 3 (2.4) | 15 (11.9) | 41 (32.5) | 67 (53.2) |  |
| Overweight  (23.0 - 27.4) | 331 (41.4) | 42 (12.7) | 75 (22.7) | 102 (30.8) | 112 (33.8) |  |
| Obese  (> 27.5) | 330 (41.3) | 149 (45.2) | 101 (30.6) | 55 (16.7) | 25 (7.6) |  |
| **Smoking Status** |  |  |  |  |  | < 0.001* |
| Yes | 124 (15.5) | 15 (12.1) | 19 (15.3) | 37 (29.8) | 53 (42.7) |  |
| No | 676 (84.5) | 179 (26.5) | 172 (25.4) | 164 (24.3) | 161 (23.8) |  |
| **Drinking Status** |  |  |  |  |  | 0.099 |
| Yes | 47 (5.9) | 9 (19.1) | 8 (17) | 9 (19.1) | 21 (44.7) |  |
| No | 553 (69.1) | 110 (19.9) | 141 (25.5) | 147 (26.6) | 155 (28) |  |
| Unknown | 200 (25) | 75 (37.5) | 42 (21) | 45 (22.5) | 38 (19) |  |
| **Developed Incident T2DM** | |  |  |  |  | < 0.001* |
| Yes | 203 (25.4) | 76 (37.4) | 43 (21.2) | 46 (22.7) | 38 (18.7) |  |
| No | 597 (74.6) | 118 (19.8) | 148 (24.8) | 155 (26) | 176 (29.5) |  |
|  | **Overall Others** | **Other ethnicities, by Cre/BW quartiles** | | | | |
|  |  | **Q1 < 0.8943)** | **Q2 (0.8943 ≤ to < 1.0952)** | **Q3 (1.0952 ≤ to < 1.3208)** | **Q4 ≥ 1.3208)** | ***P*-value** |
| Count (*n*) | 450 | 107 | 122 | 117 | 104 |  |
| Age (years) | 48.88 ± 7.51 | 45.65 ± 6.1 | 48.44 ± 7.26 | 48.86 ± 7.38 | 52.71 ± 7.63 | < 0.001* |
| Height (cm) | 156.62 ± 7.57 | 156.57 ± 7.81 | 156.15 ± 8.26 | 156.07 ± 7.45 | 157.84 ± 6.48 | 0.283 |
| Weight (kg) | 65.82 ± 13 | 75.02 ± 12.82 | 66.41 ± 12.37 | 62.99 ± 11.2 | 58.86 ± 9.99 | < 0.001* |
| WHR | 0.86 ± 0.08 | 0.86 ± 0.07 | 0.87 ± 0.08 | 0.86 ± 0.08 | 0.85 ± 0.07 | 0.737 |
| HDL Cholesterol (mmol/L) | 1.37 ± 0.38 | 1.41 ± 0.35 | 1.35 ± 0.36 | 1.37 ± 0.42 | 1.36 ± 0.38 | 0.729 |
| LDL Cholesterol (mmol/L) | 3.4 ± 0.9 | 3.47 ± 0.95 | 3.38 ± 0.83 | 3.4 ± 0.93 | 3.34 ± 0.91 | 0.767 |
| Triglycerides (mmol/L) | 1.53 ± 0.96 | 1.43 ± 0.79 | 1.48 ± 0.78 | 1.76 ± 1.31 | 1.44 ± 0.79 | 0.032* |
| Systolic Blood Pressure (mmHg) | 133.58 ± 19.23 | 130.87 ± 18.39 | 132.64 ± 19.63 | 134.7 ± 19.77 | 136.24 ± 18.8 | 0.186 |
| Diastolic Blood Pressure (mmHg) | 78.71 ± 10.78 | 78.08 ± 10.92 | 78.24 ± 10.97 | 79.27 ± 10.4 | 79.3 ± 10.92 | 0.747 |
| HbA1C (%) | 5.52 ± 0 | 5.52 ± 0 | 5.52 ± 0 | 5.52 ± 0 | 5.52 ± 0 | 1.000 |
| TC (mmol/L) | 5.43 ± 1.05 | 5.49 ± 1.04 | 5.41 ± 1.07 | 5.49 ± 1.06 | 5.34 ± 1.02 | 0.676 |
| FPG (mmol/L) | 5.3 ± 0.44 | 5.33 ± 0.47 | 5.25 ± 0.43 | 5.31 ± 0.43 | 5.33 ± 0.42 | 0.508 |
| Waist Circumference (cm) | 84.15 ± 11.12 | 90.73 ± 10.05 | 85.59 ± 11.07 | 82.07 ± 9.62 | 78.04 ± 9.84 | < 0.001* |
| BMI (kg/m²) | 26.71 ± 4.45 | 30.38 ± 4.25 | 27.11 ± 3.69 | 25.78 ± 3.68 | 23.57 ± 3.38 | < 0.001* |
| Cre (umol/L) | 71.84 ± 17.87 | 56.48 ± 9.32 | 66.46 ± 12.21 | 74.77 ± 14.53 | 90.68 ± 15.57 | < 0.001* |
| **Gender** |  |  |  |  |  | < 0.001* |
| Male | 185 (41.1) | 17 (9.2) | 35 (18.9) | 53 (28.6) | 80 (43.2) |  |
| Female | 265 (58.9) | 90 (34) | 87 (32.8) | 64 (24.2) | 24 (9.1) |  |
| **Abdominal Obesity** |  |  |  |  |  | < 0.001* |
| Yes | 99 (22) | 54 (50.5) | 33 (27) | 12 (10.3) | 0 (0) |  |
| No | 351 (78) | 53 (49.5) | 89 (73) | 105 (89.7) | 104 (100) |  |
| **Obesity (kg/m^2^)** |  |  |  |  |  | < 0.001* |
| Underweight  (< 18.5) | 6 (1.3) | 0 (0) | 0 (0) | 1 (16.7) | 5 (83.3) |  |
| Normal  (18.5 - 22.9) | 76 (16.9) | 0 (0) | 14 (18.4) | 22 (28.9) | 40 (52.6) |  |
| Overweight  (23.0 - 27.4) | 181 (40.2) | 25 (13.8) | 51 (28.2) | 58 (32) | 47 (26) |  |
| Obese  (> 27.5) | 187 (41.6) | 82 (43.9) | 57 (30.5) | 36 (19.3) | 12 (6.4) |  |
| **Smoking Status** |  |  |  |  |  | < 0.001* |
| Yes | 113 (25.1) | 8 (7.1) | 23 (20.4) | 36 (31.9) | 46 (40.7) |  |
| No | 337 (74.9) | 99 (29.4) | 99 (29.4) | 81 (24) | 58 (17.2) |  |
| **Drinking Status** |  |  |  |  |  | 0.008* |
| Yes | 21 (4.7) | 0 (0) | 3 (14.3) | 9 (42.9) | 9 (42.9) |  |
| No | 326 (72.4) | 77 (23.6) | 91 (27.9) | 84 (25.8) | 74 (22.7) |  |
| Unknown | 103 (22.9) | 30 (29.1) | 28 (27.2) | 24 (23.3) | 21 (20.4) |  |
| **Developed Incident T2DM** | |  |  |  |  | 0.492 |
| Yes | 103 (22.9) | 30 (29.1) | 28 (27.2) | 24 (23.3) | 21 (20.4) |  |
| No | 347 (77.1) | 77 (22.2) | 94 (27.1) | 93 (26.8) | 83 (23.9) |  |

Data are given as mean ± standard deviation (SD), or frequency (percentage), as appropriate. **P* < 0.05. BMI, body mass index; Cre/BW, creatinine-to-body weight; HbA1c, haemoglobin A1c; HDL-C, high-density lipoprotein cholesterol; LDL-C, low-density lipoprotein cholesterol; T2DM, type 2 diabetes mellitus.
