# Supplementary material for: Linking Creatinine‐to‐Body Weight Ratio With Diabetes Incidence: A Multiethnic Malaysian Cohort Study
Source: J Diabetes. 2025 Jan 22;17(1):e70039. doi: 10.1111/1753-0407.70039 (PMC11753918; doi:10.1111/1753-0407.70039)
Supplement: Supplementary file 4 — Table S3. The relationship between Cre/BW ratio and incident diabetes in unadjusted and adjusted proportional hazards models in overall participants. [file JDB-17-e70039-s005.docx]

**Supplementary Table S3** The relationship between Cre/BW ratio and incident diabetes in unadjusted and adjusted proportional hazards models in overall participants

|  | **Overall** | | | | | |
| --- | --- | --- | --- | --- | --- | --- |
|  | **Model 1** |  | **Model 2** |  | **Model 3** |  |
|  | **HR (95% CI)** | ***P*-value** | **HR (95% CI)** | ***P*-value** | **HR (95% CI)** | ***P*-value** |
| **Cre/BW ratio** | 0.403 | < 0.001* | 0.862 | 0.549 | 0.524 | < 0.001* |
|  | (0.315, 0.515) |  | (0.531, 1.4) |  | (0.402, 0.685) |  |
| **Cre/BW ratio quartiles** | | | | | | |
| **Q1** | **Ref** |  | **Ref** |  | **Ref** |  |
| **Q2** | 0.673 | < 0.001* | 0.682 | 0.001* | 0.755 | 0.018* |
|  | (0.557, 0.812) |  | (0.543, 0.857) |  | (0.598, 0.952) |  |
| **Q3** | 0.527 | < 0.001* | 0.503 | < 0.001* | 0.575 | < 0.001* |
|  | (0.433, 0.642) |  | (0.375, 0.674) |  | (0.425, 0.779) |  |
| **Q4** | 0.481 | < 0.001* | 0.386 | < 0.001* | 0.483 | 0.001* |
|  | (0.392, 0.589) |  | (0.251, 0.594) |  | (0.309, 0.757) |  |

Details of adjustments: Model 1 (crude), Model 2 (age, gender, ethnicity), and Model 3 (age, gender, ethnicity, waist-to-hip ratio, high-density lipoprotein cholesterol, triglyceride, systolic blood pressure). CI, confidence interval; Cre/BW, creatinine-to-body weight; HR, hazard ratio; Q, Quartile; Ref, reference.
