# Supplementary material for: Linking Creatinine‐to‐Body Weight Ratio With Diabetes Incidence: A Multiethnic Malaysian Cohort Study
Source: J Diabetes. 2025 Jan 22;17(1):e70039. doi: 10.1111/1753-0407.70039 (PMC11753918; doi:10.1111/1753-0407.70039)
Supplement: Supplementary file 5 — Table S4. The relationship between Cre/BW ratio and incident diabetes in unadjusted and adjusted proportional hazards models, stratified based on gender. [file JDB-17-e70039-s006.docx]

**Supplementary Table S4** The relationship between Cre/BW ratio and incident diabetes in unadjusted and adjusted proportional hazards models, stratified based on gender

|  | **Male** | | | | | |
| --- | --- | --- | --- | --- | --- | --- |
|  | **Model 1** |  | **Model 2** |  | **Model 3** |  |
|  | **HR (95% CI)** | ***P*-value** | **HR (95% CI)** | ***P*-value** | **HR (95% CI)** | ***P*-value** |
| **Cre/BW ratio** | 0.359 | < 0.001* | 0.61 | 0.028* | 0.712 | 0.142 |
|  | (0.258, 0.499) |  | (0.393, 0.948) |  | (0.452, 1.12) |  |
| **Cre/BW ratio quartiles** | | | | | | |
| **Q1** | **Ref** |  | **Ref** |  | **Ref** |  |
| **Q2** | 0.557 | < 0.001* | 0.724 | 0.006* | 0.7 | 0.003* |
|  | (0.422, 0.735) |  | (0.575, 0.912) |  | (0.555, 0.884) |  |
| **Q3** | 0.447 | < 0.001* | 0.718 | 0.035* | 0.652 | 0.007* |
|  | (0.333, 0.598) |  | (0.528, 0.978) |  | (0.477, 0.892) |  |
| **Q4** | 0.392 | < 0.001* | 0.717 | 0.117 | 0.679 | 0.071 |
|  | (0.293, 0.525) |  | (0.473, 1.087) |  | (0.445, 1.034) |  |
|  | **Female** | | | | | |
|  | **Model 1** |  | **Model 2** |  | **Model 3** |  |
|  | **HR (95% CI)** | ***P*-value** | **HR (95% CI)** | ***P*-value** | **HR (95% CI)** | ***P*-value** |
| **Cre/BW ratio** | 0.216 | < 0.001* | 0.412 | < 0.001* | 0.441 | < 0.001* |
|  | (0.146, 0.32) |  | (0.348, 0.558) |  | (0.349, 0.559) |  |
| **Cre/BW ratio quartiles** | | | | | | |
| **Q1** | **Ref** |  | **Ref** |  | **Ref** |  |
| **Q2** | 0.621 | < 0.001* | 0.71 | 0.004* | 0.766 | 0.028* |
|  | (0.482, 0.801) |  | (0.561, 0.899) |  | (0.604, 0.971) |  |
| **Q3** | 0.402 | < 0.001* | 0.561 | < 0.001* | 0.586 | < 0.001* |
|  | (0.306, 0.529) |  | (0.429, 0.733) |  | (0.449, 0.767) |  |
| **Q4** | 0.362 | < 0.001* | 0.423 | < 0.001* | 0.443 | < 0.001* |
|  | (0.271, 0.484) |  | (0.291, 0.614) |  | (0.305, 0.643) |  |

Details of adjustments: Model 1 (crude), Model 2 (age, ethnicity), Model 3 for male (age, ethnicity, waist-to-hip ratio, triglyceride, fasting plasmid glucose, body mass index) and Model 3 for female (age, ethnicity, triglyceride, systolic blood pressure). CI, confidence interval; Cre/BW, creatinine-to-body weight; HR, hazard ratio; Q, Quartile; Ref, reference.
