# Supplementary material for: Linking Creatinine‐to‐Body Weight Ratio With Diabetes Incidence: A Multiethnic Malaysian Cohort Study
Source: J Diabetes. 2025 Jan 22;17(1):e70039. doi: 10.1111/1753-0407.70039 (PMC11753918; doi:10.1111/1753-0407.70039)
Supplement: Supplementary file 6 — Table S5. The relationship between Cre/BW ratio and incident diabetes in unadjusted and adjusted proportional hazards models, stratified based on ethnicity. [file JDB-17-e70039-s004.docx]

**Supplementary Table S5** The relationship between Cre/BW ratio and incident diabetes in unadjusted and adjusted proportional hazards models, stratified based on ethnicity

|  | **Malay** | | | | | |
| --- | --- | --- | --- | --- | --- | --- |
|  | **Model 1** |  | **Model 2** |  | **Model 3** |  |
|  | **HR (95% CI)** | ***P*-value** | **HR (95% CI)** | ***P*-value** | **HR (95% CI)** | ***P*-value** |
| **Cre/BW ratio** | 0.45 | < 0.001* | 0.722 | 0.215 | 0.816 | 0.218 |
|  | (0.307, 0.659) |  | (0.432, 1.208) |  | (0.59, 1.128) |  |
| **Cre/BW ratio quartiles** | | | | | | |
| **Q1** | **Ref** |  | **Ref** |  | **Ref** |  |
| **Q2** | 0.689 | < 0.001* | 0.671 | 0.001* | 0.834 | 0.140 |
|  | (0.573, 0.829) |  | (0.534, 0.845) |  | (0.654, 1.062) |  |
| **Q3** | 0.514 | < 0.001* | 0.469 | < 0.001* | 0.625 | 0.005* |
|  | (0.423, 0.624) |  | (0.345, 0.637) |  | (0.449, 0.869) |  |
| **Q4** | 0.479 | < 0.001* | 0.388 | < 0.001* | 0.523 | 0.012* |
|  | (0.386, 0.594) |  | (0.243, 0.618) |  | (0.315, 0.869) |  |
|  | **Chinese** | | | | | |
|  | **Model 1** |  | **Model 2** |  | **Model 3** |  |
|  | **HR (95% CI)** | ***P*-value** | **HR (95% CI)** | ***P*-value** | **HR (95% CI)** | ***P*-value** |
| **Cre/BW ratio** | 0.253 | < 0.001* | 0.519 | 0.005* | 0.646 | 0.069 |
|  | (0.143, 0.446) |  | (0.329, 0.82) |  | (0.404, 1.034) |  |
| **Cre/BW ratio quartiles** | | | | | | |
| **Q1** | **Ref** |  | **Ref** |  | **Ref** |  |
| **Q2** | 0.679 | < 0.001* | 0.726 | 0.006* | 0.743 | 0.011* |
|  | (0.561, 0.822) |  | (0.579, 0.911) |  | (0.591, 0.934) |  |
| **Q3** | 0.537 | < 0.001* | 0.575 | < 0.001* | 0.602 | 0.001* |
|  | (0.438, 0.659) |  | (0.433, 0.763) |  | (0.452, 0.802) |  |
| **Q4** | 0.516 | < 0.001* | 0.549 | 0.002* | 0.571 | 0.005* |
|  | (0.425, 0.626) |  | (0.374, 0.808) |  | (0.386, 0.847) |  |
|  |  |  |  |  |  |  |
|  |  |  |  |  |  |  |
|  |  |  |  |  |  |  |
|  |  |  |  |  |  |  |
|  |  |  |  |  |  |  |
|  |  |  |  |  |  |  |
|  | **Indian** | | | | | |
|  | **Model 1** |  | **Model 2** |  | **Model 3** |  |
|  | **HR (95% CI)** |  | **HR (95% CI)** |  | **HR (95% CI)** |  |
| **Cre/BW ratio** | 0.581 | 0.019* | 0.725 | 0.207 | 0.863 | 0.573 |
|  | (0.369, 0.915) |  | (0.441, 1.194) |  | (0.519, 1.438) |  |
| **Cre/BW ratio quartiles** | | | | | | |
| **Q1** | **Ref** |  | **Ref** |  | **Ref** |  |
| **Q2** | 0.707 | < 0.001* | 0.694 | 0.002* | 0.736 | 0.010* |
|  | (0.582, 0.859) | < 0.001* | (0.551, 0.874) |  | (0.583, 0.929) |  |
| **Q3** | 0.532 | < 0.001* | 0.495 | < 0.001* | 0.529 | < 0.001* |
|  | (0.437, 0.649) |  | (0.371, 0.661) |  | (0.395, 0.709) |  |
| **Q4** | 0.475 | < 0.001* | 0.391 | < 0.001* | 0.428 | < 0.001* |
|  | (0.389, 0.579) |  | (0.255, 0.599) |  | (0.277, 0.661) |  |
|  | **Others** | | | | | |
|  | **Model 1** |  | **Model 2** |  | **Model 3** |  |
|  | **HR (95% CI)** |  | **HR (95% CI)** |  | **HR (95% CI)** |  |
| **Cre/BW ratio** | 0.562 | 0.076 | 0.689 | 0.147 | 0.816 | 0.218 |
|  | (0.297, 1.062) |  | (0.416, 1.14) |  | (0.59, 1.128) |  |
| **Cre/BW ratio quartiles** | | | | | | |
| **Q1** | **Ref** |  | **Ref** |  | **Ref** |  |
| **Q2** | 0.625 | < 0.001* | 0.6 | < 0.001* | 0.728 | 0.009* |
|  | (0.523, 0.745) |  | (0.478, 0.753) |  | (0.573, 0.924) |  |
| **Q3** | 0.533 | < 0.001* | 0.486 | < 0.001* | 0.616 | 0.005* |
|  | (0.437, 0.651) |  | (0.354, 0.669) |  | (0.439, 0.865) |  |
| **Q4** | 0.476 | < 0.001* | 0.396 | < 0.001* | 0.502 | 0.008* |
|  | (0.377, 0.6) |  | (0.246, 0.635) |  | (0.3, 0.837) |  |

Details of adjustments: Model 1 (crude), Model 2 (age, gender), Model 3 for Malay (age, gender, high density lipoprotein, diastolic blood pressure, waist circumference), Model 3 for Chinese (age, gender, high density lipoprotein, diastolic blood pressure), Model 3 for Indian (age, gender, high density lipoprotein, diastolic blood pressure), and Model 3 for other ethnicities (age, gender, high density lipoprotein, diastolic blood pressure, waist circumference). CI, confidence interval; Cre/BW, creatinine-to-body weight; HR, hazard ratio; Q, Quartile; Ref, reference.
