# Supplementary material for: Linking Creatinine‐to‐Body Weight Ratio With Diabetes Incidence: A Multiethnic Malaysian Cohort Study
Source: J Diabetes. 2025 Jan 22;17(1):e70039. doi: 10.1111/1753-0407.70039 (PMC11753918; doi:10.1111/1753-0407.70039)
Supplement: Supplementary file 7 — Table S6. The relationship between BMI, waist/height and incident diabetes in unadjusted and adjusted proportional hazards models in overall participants. [file JDB-17-e70039-s001.docx]

**Supplementary Table S6** The relationship between BMI, waist/height and incident diabetes in unadjusted and adjusted proportional hazards models in overall participants

|  | **Overall** | | | | | |
| --- | --- | --- | --- | --- | --- | --- |
|  | **Model 1** |  | **Model 2** |  | **Model 3** |  |
|  | **HR (95% CI)** | ***P*-value** | **HR (95% CI)** | ***P*-value** | **HR (95% CI)** | ***P*-value** |
| **Cre/BW ratio** | 0.403 | < 0.001* | 0.862 | 0.549 | 0.524 | < 0.001* |
|  | (0.315, 0.515) |  | (0.531, 1.4) |  | (0.402, 0.685) |  |
| **BMI** | 1.110 | < 0.001* | 1.047 | 0.001* | 1.026 | 0.059 |
|  | (1.096, 1.124) |  | (1.019, 1.076) |  | (0.999, 1.055) |  |
| **Waist/height ratio** | 3114.343 | < 0.001* | 26.241 | 0.001* | 14.202 | 0.007* |
|  | (1319.792, 7348.987) |  | (3.882, 177.373) |  | (2.092, 96.418) |  |

Details of adjustments: Model 1 (crude), Model 2 (age, gender, ethnicity), and Model 3 (age, gender, ethnicity, waist-to-hip ratio, high-density lipoprotein cholesterol, triglyceride, systolic blood pressure). CI, confidence interval; BMI, body mass index; HR, hazard ratio.
