# Supplementary material for: Linking Creatinine‐to‐Body Weight Ratio With Diabetes Incidence: A Multiethnic Malaysian Cohort Study
Source: J Diabetes. 2025 Jan 22;17(1):e70039. doi: 10.1111/1753-0407.70039 (PMC11753918; doi:10.1111/1753-0407.70039)
Supplement: Supplementary file 8 — Table S7. The relationship between Cre/BW ratio, interaction with ethnicity and incident diabetes in unadjusted and adjusted proportional hazards models in overall participants. [file JDB-17-e70039-s008.docx]

**Supplementary Table S7** The relationship between Cre/BW ratio, interaction with ethnicity and incident diabetes in unadjusted and adjusted proportional hazards models in overall participants

|  | **Overall** | | | |
| --- | --- | --- | --- | --- |
|  | **Model 5** |  | **Model 6** |  |
|  | **HR (95% CI)** | ***P*-value** | **HR (95% CI)** | ***P*-value** |
| **Cre/BW ratio** | 0.403 | < 0.001* | 0.862 | 0.549 |
|  | (0.315, 0.515) |  | (0.531, 1.4) |  |
| **Ethnicity** | | | | |
| **Malay** | **Ref** |  | **Ref** |  |
| **Chinese** | 0.942 | 0.861 | 1.075 | 0.836 |
|  | (0.486, 1.826) |  | (0.54, 2.143) |  |
| **Indian** | 1.171 | 0.580 | 1.304 | 0.376 |
|  | (0.668, 2.053) |  | (0.724, 2.348) |  |
| **Others** | 0.496 | 0.074 | 0.497 | 0.095 |
|  | (0.23, 1.071) |  | (0.218, 1.128) |  |
| **Cre/BW x Ethnicity (Interaction)** | | | | |
| **Cre/BW x Chinese** | 0.512 | < 0.001* | 0.607 | < 0.001* |
|  | (0.423, 0.619) |  | (0.5, 0.736) |  |
| **Cre/BW x Indian** | 1.709 | < 0.001* | 1.507 | < 0.001* |
|  | (1.432, 2.04) |  | (1.258, 1.805) |  |
| **Cre/BW x Others** | 0.579 | < 0.001* | 0.515 | < 0.001* |
|  | (0.466, 0.72) |  | (0.412, 0.643) |  |
|  |  |  |  |  |
| **Cre/BW ratio quartiles** | | | | |
| **Q1** | **Ref** |  | **Ref** |  |
| **Q2** | 0.673 | < 0.001* | 0.682 | 0.001* |
|  | (0.557, 0.812) |  | (0.543, 0.857) |  |
| **Q3** | 0.527 | < 0.001* | 0.503 | < 0.001* |
|  | (0.433, 0.642) |  | (0.375, 0.674) |  |
| **Q4** | 0.481 | < 0.001* | 0.386 | < 0.001* |
|  | (0.392, 0.589) |  | (0.251, 0.594) |  |
|  | | | | |
| **Ethnicity** | | | | |
| **Malay** | **Ref** |  | **Ref** |  |
| **Chinese** | 0.856 | 0.649 | 0.902 | 0.765 |
|  | (0.438, 1.672) |  | (0.459, 1.773) |  |
| **Indian** | 1.309 | 0.320 | 1.249 | 0.423 |
|  | (0.77, 2.226) |  | (0.725, 2.151) |  |
| **Others** | 0.543 | 0.096 | 0.469 | 0.055 |
|  | (0.265, 1.114) |  | (0.217, 1.016) |  |
| **Cre/BW x Ethnicity (Interaction)** | | | | |
| **Cre/BW x Chinese** | 0.508 | < 0.001* | 0.607 | < 0.001* |
|  | (0.42, 0.614) |  | (0.5, 0.737) |  |
| **Cre/BW x Indian** | 1.662 | < 0.001* | 1.491 | < 0.001* |
|  | (1.396, 1.977) |  | (1.248, 1.782) |  |
| **Cre/BW x Others** | 0.573 | < 0.001* | 0.521 | < 0.001* |
|  | (0.461, 0.711) |  | (0.418, 0.65) |  |

Details of adjustments: Model 5 (age, gender, ethnicity, interaction between Cre/BW ratio and ethnicity), and Model 6 (age, gender, ethnicity, waist-to-hip ratio, high-density lipoprotein cholesterol, triglyceride, systolic blood pressure, interaction between Cre/BW ratio and ethnicity). CI, confidence interval; Cre/BW, creatinine-to-body weight; HR, hazard ratio; Q, Quartile; Ref, reference.
